# Multiscale neural signatures of major depressive, anxiety, and stress-related disorders

**DOI:** 10.1073/pnas.2204433119

**Published:** 2022-06-01

**Authors:** Peter Zhukovsky, Michael Wainberg, Milos Milic, Shreejoy J. Tripathy, Benoit H. Mulsant, Daniel Felsky, Aristotle N. Voineskos

**Affiliations:** ^a^Campbell Family Mental Health Research Institute, Centre for Addiction and Mental Health, Toronto, ON M5T 1R8, Canada;; ^b^Department of Psychiatry, Temerty Faculty of Medicine, University of Toronto, Toronto, ON M5T 1R8, Canada;; ^c^Department of Physiology, University of Toronto, Toronto, ON M5S 1A8, Canada;; ^d^Institute of Medical Science, Temerty Faculty of Medicine, University of Toronto, Toronto, ON M5S 1A8, Canada;; ^e^Dalla Lana School of Public Health, University of Toronto, Toronto, ON M5S 1A8, Canada

**Keywords:** major depressive disorder, anxiety, stress-related disorders, cognitive function, functional connectivity

## Abstract

The extent of shared and distinct neural mechanisms underlying major depressive disorder (MDD), anxiety, and stress-related disorders is still unclear. We compared the neural signatures of these disorders in 5,405 UK Biobank patients and 21,727 healthy controls. We found the greatest case–control differences in resting-state functional connectivity and cortical thickness in MDD, followed by anxiety and stress-related disorders. Neural signatures for MDD and anxiety disorders were highly concordant, whereas stress-related disorders showed a distinct pattern. Controlling for cross-disorder genetic risk somewhat decreased the similarity between functional neural signatures of stress-related disorders and both MDD and anxiety disorders. Among cases and healthy controls, reduced within-network and increased between-network frontoparietal and default mode connectivity were associated with poorer cognitive performance (processing speed, attention, associative learning, and fluid intelligence). These results provide evidence for distinct neural circuit function impairments in MDD and anxiety disorders compared to stress disorders, yet cognitive impairment appears unrelated to diagnosis and varies with circuit function.

## Significance

Major depressive, anxiety, and stress-related disorders are highly comorbid and may affect similar neurocircuitry and cognitive processes. However, the neurocircuitry underlying shared dimensions of cognitive impairment is unclear and holds the promise of reimagining psychiatric nosology. Here we leverage population imaging data (*n* = 27,132) to show that while major depressive and anxiety disorders share functional and structural neural signatures, stress-related disorders are distinct from these two conditions. We report that better cognitive function is associated with lower connectivity of specific nodes of the default mode and frontoparietal networks. These findings provide population benchmarks for brain–cognition associations in healthy participants and those with lifetime major depressive and anxiety disorders, advancing our understanding of intrinsic brain networks underlying cognitive dysfunction.

Major depressive disorder (MDD) and anxiety disorders (i.e., generalized anxiety disorder and panic disorder without agoraphobia) are highly comorbid psychiatric disorders (1–3), with shared epidemiologic, developmental, and genetic features (4, 5), and are among the leading causes of disability worldwide (6). Depression and anxiety are often triggered by stressful life events, thus sharing the etiology of stress-related disorders that are defined by occurrence of a severe stressor or trauma (7). More specifically, posttraumatic stress disorder (PTSD) is characterized by hyperarousal states during recurring flashbacks to the stressful event, while stress adjustment disorder is characterized by depressive symptoms in response to a severe stressor (7). Unlike MDD and anxiety disorders, which are recurrent or chronic, stress adjustment disorder resolves within 6 mo after termination of the stressor. While considerable neurobiological research has been conducted at a disorder-specific level, few studies have investigated a broad spectrum of MDD, anxiety, and stress disorders to examine shared and distinct neural correlates.

Task-based functional MRI (fMRI) findings point to disrupted emotional processing and executive dysfunction, exemplified by disrupted cognitive control (8, 9), across a variety of disorders, including but not limited to MDD and anxiety disorders. Similarly, gray matter reductions have been shown in the insular and anterior cingulate cortices across mood, anxiety, and other disorders (10, 11). Many of these similarities in brain structure have been shown to be partially attributable to similarities in common variant architectures (12), encouraging the consideration of genetic risk measures in studies of intermediate imaging phenotypes for psychiatric illness. Polygenic liability for psychiatric disorders can be quantified using polygenic risk scores (PRSs), which are predictive of disease progression (13) and often transdiagnostically informative (14, 15). Therefore, parsing transdiagnostic phenotypes that capture the shared and distinct genetic, neurobiological, and cognitive basis of symptoms presenting across disorders could have utility in improving psychiatric nosology (16).

Inferior prefrontal and insular cortex, the inferior parietal lobule, and the putamen are hypoactivated in task-based fMRI paradigms across MDD, anxiety disorders, and stress-related disorders (17), implicating inhibitory control and salience processing as shared neural phenotypes underlying these disorders. Impairments in executive functions such as inhibitory control over emotional reactivity and negative mood may capture a transdiagnostic dimension of psychopathology (18, 19). Executive function is also impaired by anxiety, which reduces cognitive flexibility, working memory (20), and attentional control (21). While some evidence also supports executive dysfunction in PTSD (22, 23), psychological theories of posttraumatic stress typically emphasize the effects of the traumatic event on memory (24, 25). Executive dysfunction may be linked to both dysregulated mood in MDD and heightened emotional reactivity in anxiety disorders (26) and thus provides a promising transdiagnostic treatment target.

Here we leveraged multimodal data from the UK Biobank to determine unique and shared features of brain structure and function in MDD, anxiety disorders (ANX), and stress-related disorders (STR), as well as the relationship of such neuroimaging measures to several aspects of cognitive function across these disorders. The UK Biobank includes midlife and older adults and is thus also suitable for investigating cognition in the context of aging with and without MDD, anxiety, and stress-related disorders. We selected trail making performance (27), digit–symbol substitution (28), fluid intelligence (29), and paired associate learning (PAL) (30) to measure key domains of cognitive function, expecting executive function and processing speed impairments in MDD and anxiety disorders and memory deficits in stress-related disorders. We used second-order statistical comparisons to investigate genetic and environmental contributions to disorder similarity. We expected to find default mode and frontostriatal connectivity differences in MDD (31) and anxiety disorders (32, 33), resulting in shared neural signatures. We also hypothesized reduced cortical thickness of the frontoparietal regions in both MDD and anxiety disorders (34–36) and a separate neural signature of stress-related disorders focused on the hippocampal regions (37). Given varying degrees of shared genetic liability for our selected disorders and our interest in disentangling their overlapping vs. discrete neural signatures, we anticipated that controlling for disease-specific PRS would impact the correlation of cross-disorder neural signatures, providing insight into nature vs. nurture components of these intermediate phenotypes. Finally, we expected connectivity of frontoparietal (FPN), attention, and default mode networks (DMN) to underlie cognitive performance across disorders.

## Results

Demographic and clinical information for all included UK Biobank (UKB) participants is summarized in Table 1. Clinical, neuroimaging, cognitive, and genetic data from five nonoverlapping groups (MDD-, ANX-, MDD + ANX, STR-, and healthy controls) were integrated, with additional information available in *SI Appendix*.

**Table 1.** Demographic and clinical sample characteristics

	All control	Matched control	MDD-	ANX-	MDD and ANX	STR-	Group effect	*P* value
N	21,727	5,405	3,233	664	676	832		
Age at MRI	63.8(7.5)	62.5(7.4)	62.4(7.5)	63.5(7.2)	62.5(7.7)	62.3(7.1)	3.26	0.011
No. female	10,894(50%)	3,440(64%)	2,064(64%)	409(62%)	447(66%)	520(62%)	3.5	0.474
PHQ-2 ≥2	1,705(8%)	456(8%)	977(30%)	104(16%)	231(34%)	103(12%)	818.7	<0.0001
Restlessness ≥2	3,275(15%)	872(16%)	1,182(37%)	198(30%)	306(45%)	195(23%)	630.9	<0.0001
Tiredness ≥2	8,620(40%)	2,280(42%)	2,116(65%)	354(53%)	455(67%)	423(51%)	530.5	<0.0001
Age of first MDE	—	—	34.4(14.2)	—	33.2(14.9)	—	—	
Age of last MDE			51.6(11.3)		54.6(9.9)			
Age Dx first reported			43.3(14.1)	54.9(11.8)	44.5(14.8)	49.4(9.5)		
No. of MDEs	—	—	2.44(1.8)	—	2.89(2.0)	—	—	
MDD PRS	−0.03(0.99)	−0.02(1.00)	0.15(1.01)	0.09(0.99)	0.18(1.03)	−0.04(0.98)	15.3	<0.0001
ANX PRS	−0.004(1.00)	−0.006(0.99)	0.04(1.00)	0.003(0.95)	0.11(0.99)	−0.04(0.96)	2.5	0.04
PTSD PRS	−0.001(1.01)	0.005(1.00)	0.002(0.96)	0.000(0.92)	−0.004(1.03)	−0.09(1.06)	1.2	0.31
Head motion	0.11(0.05)	0.11(0.05)	0.13(0.06)	0.13(0.07)	0.13(0.07)	0.12(0.06)	37.4	<0.0001
Medication (N)								
SSRI/SARI	471	134	891	228	353	175	1872.4	<0.0001
SNRI/NRI	46	15	119	22	74	18	357.9	<0.0001
TCA	792	207	485	164	194	180	752.2	<0.0001
MAO-I	0	0	4	0	2	1	11.6	0.02
NaSSA	23	3	107	24	70	16	354.4	<0.0001

Lifetime diagnosis of MDD (F32/F33), anxiety (F41), and stress-related disorders (F43) was used to define the groups. Control participants matched for age and sex were included in case-control comparisons, whereas all control participants were included in brain-cognition analyses. Ages of first and last MDD episodes (MDE) are derived from self-report measures. Self-reported age of first MDD episode precedes the age of the first reported ICD diagnosis of MDD. Mean ages, mean PRSs, and mean head motion (±SD) are shown. Group effects were assessed using a one-way analysis of variance (F test) for age and PRSs and χ^2^ goodness-of-fit tests for categorical comparisons. Case groups were compared with the matched control group. The PHQ-2 with a cutoff score of 2 or greater was used to test for presence of depressed mood in participants at the time of scanning and cognitive testing. This threshold has high PHQ-2 sensitivity (0.91) and specificity (0.67) for diagnosis made using a semistructured interview (38). We show the total numbers of participants with lifetime use of medication falling into five categories: SSRI, selective serotonin reuptake inhibitor and SARI, serotonin antagonist and reuptake inhibitors; SNRI, selective noradrenaline reuptake inhibitor; TCA, tricyclic antidepressants; MAO-I, monoamine oxidase inhibitors; and NaSSA, noradrenergic and specific serotonergic antidepressants. Shown are MDD-, ANX-, MDD + ANX, and STR-. More information on the medications in each category can be found in *SI Appendix*, Table S4. More details on the sample sizes are available in *SI Appendix*, Fig. S14.

### Effects of Diagnosis on Neuroimaging and Cognitive Outcomes.

**Fig. 1.**
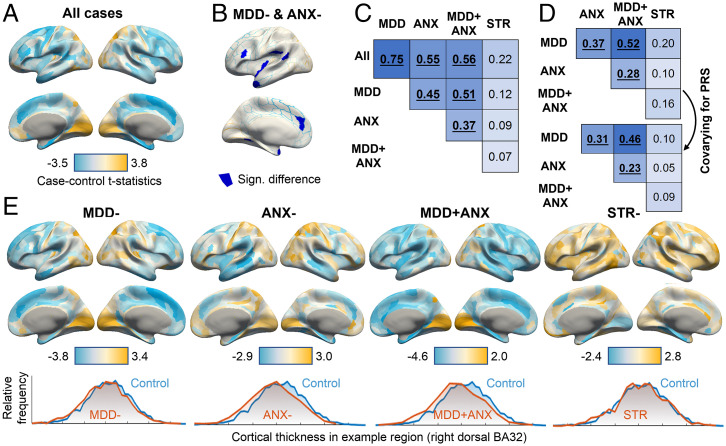
Case–control differences in cortical thickness (*A*) in all cases, and in MDD, anxiety disorders, MDD + ANX, and STR- (*E*). Distributions of cortical thickness values for each case group vs. controls for an example region of interest are plotted in *E*. Regions where a significant effect of both MDD- and ANX- groups was found are shown in blue in *B*. Effects of polygenic risk were not included as a covariate in the analysis of the full sample. Case–control t statistics of t_12,203_ = 5 correspond to an effect size d = 0.09 and t_12,203_ = 2.5 to d = 0.045. Disorder similarity matrices for the full sample (*C*) and for the unrelated White British sample (*D*) were largely consistent. Covarying for polygenic risk (PRS) slightly reduced disorder similarity (*D*). The disorder similarity matrices represent Pearson’s correlations of case–control statistics from the 360 regional cortical thicknesses. Significant correlations at P_PERM_ < 0.01 are shown in bold and underlined.

In order to compare neuroimaging signatures of MDD, anxiety, and stress-related disorders, we first calculated the case–control brain maps. Compared to healthy controls, cortical thickness was reduced across many prefrontal, parietal, and temporal regions in the MDD- and MDD + ANX group (Fig. 1*E* and *SI Appendix*, Table 3). These differences were also captured in the comparison of all cases vs. controls (Fig. 1*A*) as the case–control map for all cases was very similar to the case–control map for MDD-, ANX-, and MDD + ANX (Fig. 1 *C* and *E*). In contrast, only select temporal and parietal regions showed a significant decrease in cortical thickness in the ANX- group including middle temporal and supramarginal regions. Significantly reduced cortical thickness was also found in the parahippocampal and ventral medial visual regions of the STR- group when compared to controls.

**Fig. 2.**
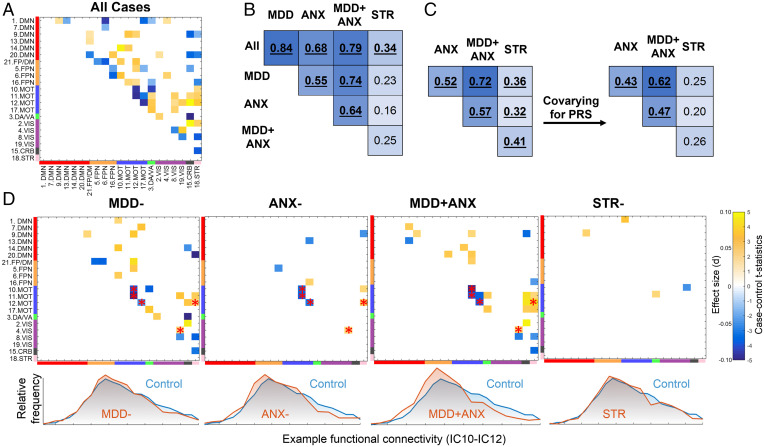
Case–control differences in functional connectivity (*A*) in all cases, and in MDD, anxiety disorders, MDD + ANX and stress-related disorders (*D*). Connectivities that showed significant differences from the control group in both MDD- and ANX- are highlighted with red asterisks (*D*). Lower half of the correlation matrix is left blank. Effects of polygenic risk were not included as a covariate in the analysis of the full sample. Disorder similarity matrices for the full sample (*B*) and for the unrelated White British sample (*C*) were highly consistent. Covarying for polygenic risk (PRS) slightly reduced disorder similarity (*C*). Disorder similarity matrices represent Pearson’s correlations of case–control statistics from the 210 connectivities between pairs of ICs. Significant correlations at P_PERM_ < 0.01 are shown in bold and underlined.

In individual comparisons of each diagnostic group to healthy controls, we identified widespread differences in resting state connectivity (Fig. 2*D*). The largest number of significant differences was found between the control and MDD- or MDD + ANX groups, whereas smaller differences were found between the control and ANX- or STR- groups. Specifically, independent component (IC)–12 (presupplementary and supplementary motor areas) showed decreased connectivity with other motor areas (IC-17, superior temporal gyrus) and increased connectivity with the striatal IC-18 across MDD-, MDD + ANX, and ANX- groups but not STR-. A comparison of all cases to controls (Fig. 2*A*) showed that the pattern of differences across all cases was very similar to the differences observed specifically in MDD-, ANX-, and MDD + ANX (Fig. 2*B*).

Case–control differences in cognitive function are summarized in Table 2 and *SI Appendix*. Compared to the control group, the four diagnostic groups showed significant impairments in all domains of cognitive function, except for visuospatial processing (trail-making test [TMT]), which was not significantly impaired in STR-, and PAL, which was not significantly impaired in MDD-, MDD + ANX, or STR-. Notably, we found impaired fluid intelligence and digit–symbol substitution performance in all case groups, suggesting a transdiagnostic pattern of impairments among these aspects of cognitive function.

**Table 2.** Cognitive performance in MDD-, ANX-, MDD + ANX, and STR-

Cognitive test	Statistic	MDD-	ANX-	MDD + ANX	STR-
TMT	Standard β	**0.06**	**0.12**	**0.19**	0.06
	T-stat	**2.33**	**2.70**	**3.98**	1.33
Gf	Standard β	**−0.05**	**−0.14**	**−0.16**	**−0.08**
	T-stat	**−2.33**	**−3.56**	**−4.03**	**−2.29**
PAL	Standard β	−0.04	**−0.13**	−0.05	−0.06
	T-stat	−1.54	**−2.91**	−1.09	−1.51
DSST	Standard β	**−0.12**	**−0.11**	**−0.28**	**−0.09**
	T-stat	**−5.20**	**−2.61**	**−6.18**	**−2.30**

TMT, visuospatial processing; Gf, fluid intelligence. Standardized beta-coefficients are shown. For instance, DSST performance in the MDD + ANX group was 0.28 SD below the performance of the control group. Higher scores on TMT indicate worse performance due to longer times to complete the task. Significant effects are shown in bold (FDR-corrected *P* < 0.05).

### Similarity of Diagnosis-Specific Neural Signatures.

After constructing diagnosis-specific case–control neural signatures, we evaluated the pairwise similarities in their spatial distributions and component effect sizes. A large degree of overlap was found between MDD-, ANX-, and MDD + ANX groups in both functional connectivity and cortical thickness case–control associations (Fig. 2 *B* and *C*). On the other hand, the STR- group showed a different spatial pattern of associations that did not resemble the other disorders (*P*_PERM_ > 0.01).

The similarities of neural signatures of the disorders represent the shared influences of both heritable and environmental factors. To determine the degree to which these similarities were moderated by interindividual differences in heritable risk for each of these comorbid disorders, we performed the same analysis again but this time including PRS for all three diagnoses as covariates. After controlling for these genetic risk profiles, correlations between diagnostic neural signatures for the MDD-, ANX-, MDD + ANX, and STR- groups were slightly reduced for both functional and structural measures (Figs. 1*D* and 2*C*). The correlations between STR- and all other diagnostic groups were affected to the greatest degree compared to other groups.

### Sensitivity Analyses.

A comparison of active MDD with the other disorder groups revealed a highly similar pattern of case–control differences to the analysis of the full sample (*SI Appendix*). Therefore, restricting the sample to active MDD did not alter the disorder similarity matrices. Focusing on nonmedicated participants also did not substantially alter the results, suggesting that our main findings are likely driven by the presence of lifetime diagnosis rather than medication.

### Neural Correlates of Cognitive Function in MDD-, ANX-, MDD + ANX, and STR-.

**Fig. 3.**
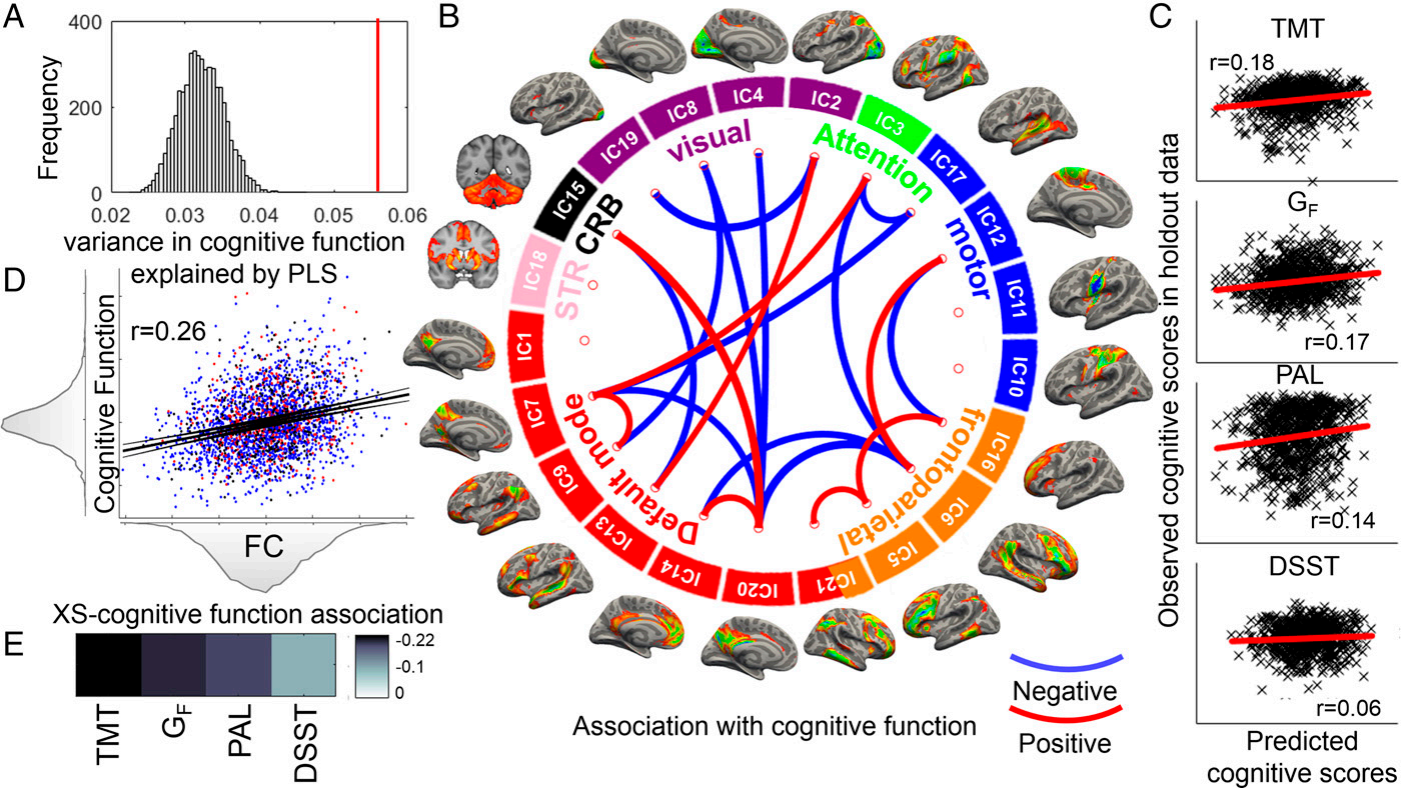
Brain–cognition relationships between functional connectivity (FC) and cognitive function from a PLS regression in participants with major depression, anxiety, or stress-related disorders. The model explained a significantly larger amount of variance (*P* < 0.001) than expected by chance (*A*). PLS latent variable 1 (PLS1) accounted for the largest amount of variance in cognitive tests (*D*). PLS1 scores for MDD- are shown in blue, MDD+ANX in red, and ANX- in black (*D*). Higher PLS1 FC scores (XS) were associated with worse cognitive performance on all four tests (*E*), characterized by longer times to complete the TMT, lower number of correct reasoning questions in the fluid intelligence test (Gf), lower number of word pairs recalled on the PAL test, and lower number of digits being filled in in the DSST. Thresholded PLS1 weights (Z > 3 and Z < −3) implicated pairwise connectivities between independent components (ICs) corresponding to the default mode, frontoparietal, and dorsal/ventral attention networks (*B*). Blue connections between network components suggest that higher connectivity of those components was associated with worse cognitive performance. Red connections between network components suggest that higher connectivity of those components predicted better cognitive performance. The PLS model was able to predict the variability in cognitive function in held-out data (*C*). Other network labels: CRB, cerebellum; STR, striatum.

Given the substantial, albeit variable, overlap in functional neural signatures between the diagnostic groups, we aimed to uncover those circuits specifically related to worse executive function and verbal memory across all disorders (*n* = 3,216) after regressing out age, sex, site, and motion confounds. Partial least squares (PLS) regression identified three latent variables that explained 3.1%, 1.5%, and 1.3% of variance in the four cognitive function tests (TMT, fluid intelligence [G_F_], PAL, and digit–symbol substitution task [DSST]). Permutation testing showed that these latent variables together explained a significant amount of variance in cognitive performance (*P*_PERM_ < 0.001; Fig. 3*A*).

The first latent variable (PLS1) representing functional connectivity, which was optimally associated with cognitive performance, captured the most variance in the outcome variables (Fig. 3*A*) and was associated with worse performance on all four cognitive tests. We found 11 connectivities with normalized PLS1 weights of Z > 3 (significant and positive) and 8 connectivities with normalized PLS1 weights with Z < −3 (significant and negative) (Fig. 3 *B* and *C*). Functional correlates of worse cognitive performance included connectivities of the frontoparietal, default mode, and attention networks. Increased DMN–FPN connectivities were also associated with worse cognitive function (e.g., IC-6 with IC-14 or IC-20 with IC-6). Decreased within-DMN (IC-20 with IC-14 or IC-7 with IC-9) and within-FPN (IC-5 with IC-21 or IC-5 with IC-16) connectivities were associated with worse cognitive function.

The second latent variable (PLS2) captured some variance in fluid intelligence and PAL (*SI Appendix*, Fig. S10). We found four connectivities with significant positive loadings on PLS2 (Z > 3) and one weight with significant negative loading (Z < −3). Higher connectivity of IC-21 (FPN/DMN) with IC-5 (FPN) and IC-1 (DMN) and higher connectivity of the striatal component (IC-18) with the superior temporal gyrus (IC-17) predicted better fluid intelligence and associative learning. Lower connectivity of IC-21 (FPN/DMN) with IC-6 (FPN) predicted lower fluid intelligence and associative learning scores. Although the third latent variable (PLS3) was significant at permutation testing, there were no PLS3 weights |Z| > 3.

**Fig. 4.**
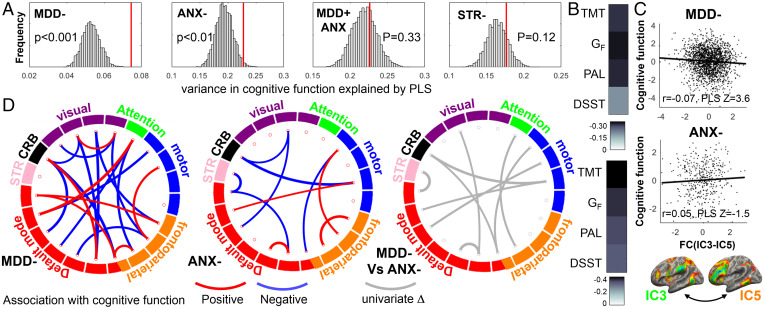
Brain–cognition relationships between functional connectivity and cognitive function from a PLS regression in MDD-, ANX-, or STR- groups. Repeating the PLS regressions in each case group separately revealed that the brain–cognition relationships were driven by MDD- and ANX- groups, with no significant relationships found in MDD + ANX or STR-. (*A*) Permutation distributions of percent of variance in cognitive function explained by the respective PLS model is shown in gray, with the observed value shown in red. (*B*) The associations between PLS1 scores in MDD- (*Upper*) and in ANX- (*Lower*) with the four cognitive function tests. (*D*) Connectivities associated with cognitive function (|Z| > 3) in MDD- and ANX- and significant differences (|Z|>1.96, uncorrected *P* < 0.05) between these two groups in connectivities associated with cognitive function identified in univariate analyses. (*C*) For instance, higher connectivity of IC-3 with IC-5 was associated with worse cognitive function measured by the first principal component of variance in the four cognitive scores in MDD-. This association was in the opposite direction and was not robust in ANX-, resulting in a significant group difference in the association between IC-3–IC-5 connectivity and cognitive function.

To contrast transdiagnostic signatures with diagnosis-specific signatures, we repeated the PLS regression in each case group separately. We found significant brain–cognition relationships in MDD- and in ANX- but not in MDD + ANX or STR- (Fig. 4 *A* and *B* and *SI Appendix*), suggesting that former two groups drive the associations across all cases. We found a distinct pattern of brain–cognition relationships in MDD- and ANX-. While components from the FPN and DMN played a major role in brain–cognition relationships in each case group, different connectivities among different components of these networks were implicated in MDD- and ANX-. Many components that were significant (|Z| > 3) in MDD- and ANX- separately were also significant in the PLS regression across all cases.

In order to directly compare brain–cognition associations in MDD- and ANX- post hoc, we used univariate Pearson’s correlations between functional connectivities and the first principal component capturing the largest amount of variance in Y (46%). We found several connectivities that were significantly different between MDD- and ANX-, each of which was significantly associated with cognitive function in one group but not in the other group (Fig. 4 *C* and *D*).

### Neural Correlates of Cognitive Function in the Control Group.

We repeated the PLS analysis in the control group. Briefly, the PLS model explained 3.6% of variance in the cognitive outcomes (P_PERM_ < 0.001; *SI Appendix*). PLS1 was also linked to worse general cognition in healthy controls. PLS1 weights included ICs from the DMN, FPN, and dorsal/ventral attention [DA/VA] network (*SI Appendix*, Figs. S11 and S12). PLS2 was also linked to worse cognition, similarly to PLS2 in the case group PLS.

## Discussion

In a multimodal investigation of functional connectivity, brain structure, cognitive function, and genetic risk, we found that signatures of MDD and anxiety disorders were highly concordant and distinct from stress-related disorders. Shared polygenic risk explained a small proportion of the similarity in brain connectivity and structure between MDD, anxiety, and stress-related disorders. We further identified impairments in processing speed, attention, and fluid intelligence shared across MDD, anxiety, and stress-related disorders. A dimensional analysis cutting across these disorders identified increased between-network and decreased within-network connectivity of the frontoparietal-default mode networks as a neural correlate of poorer cognitive function.

### Case–Control Differences.

The MDD- and MDD + ANX groups showed cortical thickness differences from the control group that were consistent with previous meta-analyses of brain structure in MDD (34, 35), with notable thinning of the parietal and prefrontal cortex (insula, anterior cingulate, inferior frontal, superior frontal and middle frontal gyri, and anterior temporal lobe). Comorbid MDD and anxiety are typically associated with greater symptom severity and more limited level of daily functioning (39, 40). Consistent with the expectation of greater disorder severity in comorbid MDD and anxiety disorders, we found additional thinning in the medial temporal regions (parahippocampal area), suggesting a more profound impact of the comorbid disorders on brain structure. The ANX- group showed localized patterns of cortical thinning in the middle temporal gyri, which is in line with previous evidence of reduced volumes of these regions in anxiety disorders without comorbid MDD (11). Unlike studies of active anxiety disorders, we did not find significant differences in the anterior cingulate or insular cortices in our ANX- group. This may be explained by the possible time gap between diagnosis and imaging data. In the UKB, lifetime diagnosis is recorded, and many participants may not be experiencing clinical symptoms at the time of scanning. One possibility is that brain volumes may have recovered since the time of formal psychiatric diagnosis. Finally, the STR- group showed few significant differences from controls, characterized primarily by localized cortical thinning in the parahippocampal area. Parahippocampal cortex is critical to memory formation and retrieval, and medial temporal areas have been emphasized in studies of PTSD (41, 42). Further, the hippocampus has been shown to regulate stress response by influencing the hypothalamic–pituitary–adrenal function (37). Therefore, our findings in the STR- group are consistent with existing theories of stress regulation in the medial temporal lobe.

Significant resting-state connectivity deviations in people with MDD and MDD with comorbid anxiety were similar and encompassed a variety of regions that were part of the default mode and frontoparietal but also subcortical (striatal), cerebellar, and visual networks. Deviations in connectivity within the motor network and between motor and striatal networks were common across the MDD, anxiety disorders, and MDD comorbid with anxiety groups. Our findings are consistent with previously reported alterations in frontoparietal and default mode connectivity in MDD (31) and in anxiety disorders (32, 33). Connectivity between striatal and cortical regions is known to be impaired in MDD (43) and was also altered in both our MDD groups. Importantly, functional connectivity was significantly different between the anxiety disorders and the control group but not in stress-related disorders, reinforcing the differences in neural signatures of this group. Although the effect sizes of case–control differences were relatively small (0.05 < d < 0.15), they were slightly larger than effects previously reported in large-scale studies of psychiatric disorders (44, 45). It has been shown that small effect sizes in large-scale samples examining brain–behavior relationships tend to be reproducible, compared to larger effects in smaller *n* studies (46). While it is possible that studying more ill patients might lead to larger effect sizes or different findings, our analyses focused on actively ill patients were similar to our overall results. Effect sizes found in the UKB likely reflect the heterogeneity of this large-scale community-recruited sample and support the investigation of neurobiological dimensions underlying this variability consistent with the research domain criteria approach (16).

Substantial evidence shows that scanner type and site are two of the most important confounds in multicenter studies (47–50). Correcting for such confounds can be challenging (47) and can decrease the effect of diagnosis. Site effects also present a challenge in the UKB data (51). Approaches used by UKB and in our analyses may have mitigated site and scanner effects. First, UKB uses identical scanner hardware and software in all three sites, although subtle differences, e.g., in the same coil equipment cannot be ruled out (51). Second, IC analysis-based denoising of resting-state fMRI data used in UKB diminishes site scan differences (52), potentially improving the power of our fMRI analyses. Third, we controlled for linear effects of site in all analyses and were able to detect moderate but robust effects.

### Similarity of Neural Disorder Signatures.

The strong similarity of the neural signatures of the MDD and anxiety disorders groups supports previous studies of shared neural signatures of mental disorders (12). Controlling for polygenic risk somewhat reduced the similarity between MDD, anxiety disorders, and stress-related disorders. High genetic correlation between MDD and anxiety disorders (5, 53) could instead account for phenotypic and clinical similarities between the disorders, and not all genetic similarity may be captured by PRSs. By contrast, stress-related disorders show less genetic similarity to MDD or anxiety disorders (5). Further, the STR- group showed very few differences from healthy controls, suggesting lack of effects rather than a different pattern of neural changes relative to controls is distinguishing STR- from MDD- and ANX-.

Several neurocognitive processes may underlie the overlap in neural signatures of MDD and anxiety disorders, including executive functioning (19). A shared neural mechanism could be found in prefrontal regulation of the default mode and limbic circuits responsible for mood and emotional processing. Stress-related disorders, on the other hand, showed differences in the parahippocampal structure and default mode connectivity that may be linked to maladaptive stress response and memory formation.

### Transdiagnostic Association of Cognitive Function with Neural Signatures.

We identified impairments in different aspects of cognitive performance, including executive function in the four diagnostic groups, consistent with previous literature suggesting that executive dysfunction may be a transdiagnostic dimension of cognitive impairment (16, 54, 55). Executive function impairments are not unique to mental disorders as physical problems, acute stress, or social isolation can also impact executive performance (56).

While much is known about neural correlates of executive function (57–61), lack of replication and small samples call for large-scale data (>2,000 individuals) to uncover reproducible brain-wide associations with cognition (46). In our large UKB sample, we found a specific functional connectivity profile between nodes of the frontoparietal, default mode, and salience/attention networks and the cerebellum as underlying worse processing speed, spatial attention, fluid intelligence, and PAL. We showed that reduced within-network and increased between-network connectivity of the frontoparietal and default mode networks predicted worse executive function and related cognitive impairments in MDD, anxiety, and stress-related disorders. A similar pattern of brain–cognition relationships was found in the healthy control sample. These findings are consistent with previous multimodal metaanalyses showing that neural correlates of executive function in clinical populations encompass frontal, parietal, and cerebellar regions (60) and previous large-scale analysis relating frontoparietal and default mode connectivity to worse verbal-numerical reasoning (61) in UKB. Successful executive functioning relies on dynamic switching between frontoparietal and default mode networks initiated by cinguloopercular attentional networks (59, 61). Self-referential processing in the default mode network is balanced with goal-oriented solving of complex problems (executive function) and influenced by salient events that can trigger the switching by attention networks.

Executive function itself is multidimensional, with processing speed, cognitive flexibility, fluid intelligence, working memory, planning, attention, and inhibitory control serving as interrelated subdimensions (56). The second latent variable identified in our PLS analysis (PLS2) uncovered functional correlates of poorer performance in fluid intelligence and associative learning. In addition to connectivity pairs already identified as related to general cognitive function by the first PLS latent variable, PLS2 showed that higher connectivity of striatal areas with superior temporal gyri predicted better fluid intelligence and associative learning specifically. Identifying robust neural correlates of cognitive function using resting-state connectivity has been challenging due to the limitations of the sample sizes (46) and the interindividual variability in brain function and its relation to cognitive performance (62, 63). Here we identify robust brain–cognition relationships that are replicable in a healthy population, furnishing evidence for existing theories of how functional organization of the brain is associated with cognitive dysfunction, including executive impairments.

We did not find that brain–cognition relationships identified across all diagnostic groups were necessarily present in each of the groups separately. Only MDD- and ANX- showed significant associations between cognitive performance on all four cognitive tests and functional connectivity captured by the first latent PLS variable. A comparison of the univariate correlations showed that some of the brain–cognition associations were distinct between these two groups. Higher striato–default mode connectivity and lower within-default mode connectivity was associated with worse cognitive function in ANX- but not in MDD-. On the other hand, higher between-network connectivity (e.g., between attention and frontoparietal components) was associated with worse cognitive function in MDD- but not in ANX-. These findings suggest that brain–cognition relationships may not be uniform across MDD and anxiety disorders. Instead, different aspects of frontoparietal and default mode interactions may underlie cognitive dysfunction in MDD and anxiety disorders.

### Limitations and Conclusions.

The cross-sectional nature of the data does not allow us to disambiguate whether the neural signatures are the consequence of or a marker of vulnerability to the respective disorder. Although diagnostic groups were defined by lifetime diagnosis, we found that approximately one-third of participants in our MDD or MDD with comorbid anxiety groups had some current depressive symptoms based on the two-item patient health questionnaire (PHQ-2) (38) at the time of cognitive and MRI assessment. We show that our results did not change when restricting the sample to active MDD. PHQ-2 with a threshold of 2 has a high sensitivity but moderate specificity of 0.67 (38) as a screening tool for depression, and many individuals in the active MDD group showed only mild symptoms. Given that the PHQ-2 was generally designed as a screening tool maximizing sensitivity at the expense of specificity, we are less able to draw inference about active illness and conclude that our findings appear to be driven by lifetime diagnosis. Restricting the sample to unmedicated participants also produced consistent findings to the full sample. However, chronicity of the psychiatric disorders may differ given that the diagnosis of MDD- was reported at a younger age compared to ANX- or STR-. Further, diagnoses were ascertained using electronic health records that may show some heterogeneity (64).

While our analysis showed a similar signature of brain structure and function deviations across MDD and anxiety disorders, there are limitations to the degree of overlap between the disorders. No significant deviations in cortical thickness were seen in anxiety disorders, while the effects on cortical thickness seen in MDD and in the comorbid group were much larger and reached statistical significance. Interestingly, neuroimaging correlations accounted for less than 50% of variance in the disorder brain maps, suggesting that there are disorder-specific abnormalities in brain function and structure (65).

In conclusion, we found a high degree of similarity in the neural signatures of MDD and anxiety disorders (alone and in comorbidity) that was distinct from stress-related disorders. Our findings are consistent with the diagnostic categorization of MDD and anxiety disorders as internalizing disorders (7). Stress-related disorders showed a very similar profile of executive dysfunction to MDD and anxiety disorders, yet their neural signatures showed less similarity, especially in the domain of cortical thickness. While the comorbidity across disorders is viewed as a therapeutic challenge, the identified neurobiological substrate of connectivity within and between default mode and frontoparietal networks subserves cognitive dysfunction and could provide a promising target for specific interventions.

## Methods

### Data.

#### Healthy controls.

Participants were recruited through the UKB. The overall number of people with UKB preprocessed MRI outputs was 40,669. Healthy control group was defined by excluding participants who had any one of over 143 International Classification of Diseases (ICD-10) codes related to conditions that could affect neural connectivity and structure such as epilepsy and Alzheimer’s disease, thus resulting in 21,727 healthy controls (*SI Appendix*).

#### Patients.

We used linked health record data available from inpatient and primary care information. ICD-10 codes F32 (a major depressive episode; UKB data field 130895), F33 (recurrent major depressive episodes, UKB data field 130897), F41 (generalized anxiety disorder, panic disorder without agoraphobia, other mixed, specified, and unspecified nonphobic anxiety disorders; data field 130907), and F43 (reaction to severe stress such as PTSD, and stress adjustment disorders; UKB data field 130911) were used to define four mutually exclusive patient groups. These groups included participants with lifetime diagnoses of 1) MDD but not anxiety or stress-related disorders (MDD-), 2) nonphobic anxiety disorders but not MDD or stress-related disorders (ANX-), 3) comorbid MDD and anxiety but not stress-related disorders (MDD + ANX), and finally, 4) stress-related disorders but not MDD or anxiety (STR-). Case definition was based on lifetime diagnoses as we wanted to investigate the neural and genetic signatures of vulnerability for and consequences of the psychiatric disorders.

Depression symptoms at the time of MRI scan were assessed using a cutoff score of 2 on the PHQ-2 (38) (UKB data fields 2050 and 2060); self-reported restlessness and tiredness indices were also included (data fields 2070 and 2080, respectively).

#### Neuroimaging data acquisition.

Briefly, MRI data were collected on Siemens 3T Skyra and 32-channel receive head coil (T1-weighted structural sequence repetition time TR = 2,000 ms, echo time TE = 2.01 ms, inversion time TI = 880 ms, flip angle = 8°, resolution = 1 mm^3^). Multiband gradient echo planar imaging sequence (length = 6 min, field of view = 210 mm, slices = 64, TR = 735 ms, TE = 39 ms, resolution = 2.4 mm^3^) was used to acquire resting-state fMRI scans. More detail on the imaging protocols can be found in ref. 66. Structural images were processed using Freesurfer (67, 68), while functional images were processed using FMRIB’s Multivariate Exploratory Linear Optimized Decomposition into Independent Components (MELODIC) and FSLnets tools (66, 69). If images were flagged as unusable by UKB’s automated quality control pipeline, they were excluded from analysis.

#### Neuroimaging processing.

Two neuroimaging modalities were included: resting-state functional connectivity and cortical thickness. We used 210 resting-state connectivity features comprising all pairs of partial correlations among the 21 ICs identified by the UKB preprocessing pipeline in an IC analysis (data field 25752). We mapped 19 of the 21 ICs that were primarily located in the neocortex to Yeo7 networks (70) by comparing the proportions of voxels in each component (thresholded at z > 3) that fell into each of the seven networks (*SI Appendix*, Table S1). One component primarily encompassed the cerebellum, while another component encompassed subcortical regions (notably the striatum).

We also used UKB-provided Freesurfer outputs (data field 20227) to derive cortical thickness values for the 360 regions in the HCP parcellation (71). HCP labels were first registered from *fsaverage* space to subject space (*mri_label2label*), and summary statistics for each label were generated (*mri_segstats*). These derivatives will be made available through the UKB Returns Catalogue (https://biobank.ctsu.ox.ac.uk/crystal/docs.cgi?id=1, Project ID 61530).

#### Cognitive data.

We used four cognitive tests assessing executive function, processing speed, and learning: time to complete the alphanumeric path of the TMT (data fields 6350 and 6351) as a proxy for visuospatial processing speed and executive function (72); fluid intelligence (data field 20016); PAL (data field 20197) as a measure of memory and associative learning (73); and the DSST (data field 23324) as a measure of attention, visuoperceptual speed, and associative learning (74). In addition to testing cognitive function, TMT and DSST also tap into participants’ motor speed as participants with worse ability to write and draw will be at a disadvantage. Cognitive test data were collected during the same visit as the MRI scan. More information can be found in *SI Appendix*.

#### Genetic data.

PRSs were derived from public genome-wide association study (GWAS) summary statistics using a standard “prune and threshold” approach. All PRS analyses were performed on a subset of participants of unrelated White British ancestry, defined using the same criteria as a previous study (75). Summary statistics for MDD (76) and PTSD (77) were obtained from the Psychiatric Genomics Consortium (https://www.med.unc.edu/pgc/download-results), while those for ANX were obtained from the Integrative Psychiatric Research consortium (78) (https://ipsych.dk/fileadmin/ipsych.dk/Downloads/daner_woautism_ad_sd8-sd6_woautismad_cleaned.gz). To avoid sample overlap with the UKB, a version of the MDD summary statistics with participants from the UKB removed (daner_pgc_mdd_meta_w2_no23andMe_rmUKBB.gz) was used; ANX and PTSD summary statistics did not have any sample overlap with the UKB.

PRSs were calculated using a previously described computational pipeline (15). Briefly, the UKB’s imputed genotypes were first quality controlled using version 2.00 of the plink GWAS analysis software (79) by filtering to autosomal, nonduplicate single-nucleotide variants with imputation information (INFO) score > 0.8, and with Hardy–Weinberg equilibrium *P* > 10^−10^, missingness < 5%, and minor allele frequency > 0.1% across self-reported White participants. Summary statistics were harmonized with these quality-controlled UKB imputed genotypes with respect to reference/alternate allele and strand using the allele harmonization framework from munge_sumstats.py in the ldsc software package (80), then thresholded to *P* < 0.05 and pruned to *r*^2^ < 0.5 using frequency-informed linkage disequilibrium pruning with a 500-kb sliding window. The remaining variants constituted the trait’s PRS, with the variants’ effect sizes (log odds) constituting the weights of the PRS. Finally, PRSs were scored on each individual in the cohort by summing, across the variants in the PRS, the variant’s weight times the individual’s number of effect alleles of that variant; missing genotypes were mean-imputed. PRSs were standardized to zero mean and unit variance across all unrelated White British participants in the UK Biobank.

Before computing associations between PRSs and MRI-derived features, the first 10 genotype principal components were regressed out of the PRS.

### Statistical Analysis.

#### Effect of diagnosis and polygenic risk on neuroimaging and cognition.

We tested for case–control differences in partial correlations between functional ICs as well as regional cortical thickness using separate general linear models (*fitlm*, *anova*, MATLAB R2016a). Sex, age, age^2^, age × sex, average head motion during the resting-state fMRI run, and UKB imaging acquisition site were included as covariates. These models were specified as follows:

$$\begin{array}{l} P\mathrm{airwise}\;\mathrm{connectivity}\;∼\;g\mathrm{rou}p_{Dx}+{\text{age}+\text{age}}^{2}+\text{age×sex}+\text{sex}+\\ \text{head}\;\text{motion}+\text{site,}\\ R\mathrm{egional}\;\mathrm{cortical}\;\mathrm{thickness}\;∼\;g\mathrm{rou}p_{Dx}+{\text{age}+\text{age}}^{2}+\text{age×sex}+\\ \text{sex}+\text{site}. \end{array}$$

We reanalyzed the data using linear models including PRS for MDD, anxiety disorders, and PTSD as covariates. This analysis allowed us to estimate the effect of heritable MDD, ANX, and PTSD risk on functional connectivity and cortical thickness measures. These models were specified as follows:

$$\begin{array}{l} P\mathrm{airwise}\;\mathrm{connectivity}\;∼\;g\mathrm{rou}p_{Dx}+{\text{PRS}}_{\text{MDD}}{+\text{PRS}}_{\text{ANX}}{+\text{PRS}}_{\text{PTSD}}+\text{age}+\\ {\text{age}}^{2}+\text{age×sex}+\text{sex}+\text{head}\;\text{motion}+\text{site,}\\ R\mathrm{egional}\;\mathrm{cortical}\;\mathrm{thickness}\;∼\;g\mathrm{rou}p_{Dx}+P{\text{RS}}_{\text{MDD}}{+\text{PRS}}_{\text{ANX}}{+\text{PRS}}_{\text{PTSD}}+\\ \text{age}+{\text{age}}^{2}+\text{age×sex}+\text{sex}+\text{site}. \end{array}$$

Permutation testing (*n* = 1,000, *P*_PERM_ < 0.05) was used to test whether diagnostic group had a significant effect (F test) on functional connectivity and cortical thickness. We permuted each outcome variable (pairwise connectivity or regional cortical thickness) and repeated the linear model with the shuffled outcome variable. A significant group effect was present when the observed F value was higher than 95% of the F values obtained from permutation models. When a significant group effect was found, each of the case groups was compared to the healthy control group. We used Bonferroni correction for the post hoc tests (*P* < 0.0125) to assess significance in addition to the permutation testing of the main effect of group.

Further, we used linear models to test for an effect of group on the cognitive function variables while covarying for sex, age, age^2^, age × sex, and testing center. Standardized beta coefficients were obtained by z-scoring cognitive data before analysis. To correct for four group comparisons across 10 tests, false discovery rate with the Benjamini–Hochberg method (q < 0.05) was used (81). For greater interpretability, we converted the resulting t statistics to standardized effect sizes (Cohen’s *d*s) via the following transformation (44):

$$d=\frac{2*\text{t}}{\sqrt{df}}.$$

Since the control group was nearly four times larger than all cases combined, we selected a random subset of healthy controls such that there was an equal sample size of cases and controls in each of the above analyses. The resulting control group was matched for sex and age to the combined case group. For each case participant, the matching algorithm first identified a pool of controls of the same sex and age, then excluded candidates in the pool who had already been included in the matched control group. Finally, a control participant was selected at random from the resulting pool of candidates. We repeated the case–control analyses comparing all cases combined in one group to controls.

#### Disorder similarity.

For each case–control comparison (i.e., all cases, MDD- vs. control, ANX- vs. control, MDD + ANX vs. control, and STR- vs. control), the above-mentioned linear models generated 210 + 360 t statistics, one for each functional connectivity and cortical thickness feature. To assess the degree of cross-disorder concordance in MRI signatures, we computed Pearson’s correlations between each pair of disorders across the 210 functional connectivity t statistics and, separately, across the 360 regional cortical thickness t statistics. The statistical significance of these correlations was assessed via permutation testing: random distributions of case–control t statistics were generated by rerunning the linear models with permuted outcome variables and generating a random distribution of correlations between these t statistics (all code available at https://github.com/peterzhukovsky/ukb_transdiagnostic). We compared disorder similarity matrices generated from the 1) case–control t statistics for the full sample; 2) t statistics for White British participants; and 3) t statistics for White British participants while covarying for their polygenic risk for MDD, anxiety, and PTSD.

#### Sensitivity analyses.

We repeated the main disorder similarity analysis first, only in active MDD and second, only in unmedicated participants to determine whether these findings held independently of illness state or medication status.

#### Neural correlates of cognitive function.

PLS regression was used to assess the relationship between functional connectivity and cognitive function at the time of the imaging visit. A separate PLS regression was used to test for multivariate associations between regional cortical thickness and cognitive performance (*SI Appendix*). Model significance was tested using permutation testing following previous studies (82). In these analyses, we focused on participants in all case groups with no missing cognitive data (*n* = 3,216). We regressed out age, sex, and site from both neuroimaging and cognitive variables; we also regressed out average head motion from the fMRI data. We used z-scored residuals from these regressions to form the predictor matrix X (3,216 × 210) and the outcome matrix Y (3,216 × 4). We repeated the PLS regression exploring brain–cognition associations in healthy controls (*n* = 14,199) and in each of the four case groups separately (*SI Appendix*).

PLS returns a set of components that attempt to maximize the covariance between the PLS scores summarizing X and Y. PLS scores are a linear combination of the predictor variables (X) and component loadings. We used bootstrapping (*n* = 5,000) to identify which predictors showed robust contributions to the PLS latent variable. A threshold of |Z| > 3 was chosen to identify the most robust connectivities associated with cognitive performance (82).

#### Robustness analysis in hold-out data.

In order to evaluate the robustness of PLS performance in all cases, we have split these participants into four subsamples. In four PLS analyses, we used three of these subsamples as training data (75%, *n* = 2,412) and the remaining subsample as test data (25%, *n* = 804). We then applied the PLS beta regression coefficients obtained in the training sample to the test sample and correlated the observed cognitive data with the predicted cognitive data to assess PLS performance in predicting cognitive function in held-out data. This approach was repeated in all controls to assess robustness in hold-out data.

#### Directly comparing brain–cognition associations.

In order to directly compare the brain–cognition associations, we first extracted the first principal component of variance in the four cognitive tests in MDD- and ANX- using a principal component analysis (PCA), effectively reducing the Y matrix to the first principal component scores, which are comparable across groups. We then computed Pearson’s correlations between each of the 210 functional connectivities and the first principal component representing cognitive outcomes in the ANX- and MDD- groups separately. We compared the r-to-z transformed Pearson’s correlations between MDD- and ANX-. Significance was determined as follows:

$$Z_{\text{observed}}=\frac{z_{\text{MDD}}-z_{\text{ANX}}}{\sqrt{\frac{1}{n_{\text{MDD}}-3}+\frac{1}{n_{\text{ANX}}-3}}},$$

where $z_{\text{MDD}}$ and $z_{\text{ANX}}$ refers to the r-to-z transformed Pearson’s correlation between a pairwise connectivity and the cognitive function PCA score and $n_{\text{MDD}}$ and $n_{\text{ANX}}$ refer to sample sizes in MDD- and ANX-, respectively. When |$Z_{\text{observed}}$| > 1.96, the univariate correlations between functional connectivity and cognition are significantly different (uncorrected *P* < 0.05).

## Supplementary Material

Supplementary File

## Data Availability

The data analyzed here are from the UK Biobank, which is a uniquely powerful biomedical database. It aims to facilitate research in life sciences by providing multiscale data for a large number of participants. The UK Biobank legally binds the researchers using the data not to publicly share UK Biobank data. Therefore, we are unable to share the data in a public repository. However, all data used here can be accessed by making a request with the UK Biobank. The UK Biobank has a dedicated portal for applying for data access here: https://www.ukbiobank.ac.uk/enable-your-research/apply-for-access. The use of UK Biobank data is not entirely free, but the data access costs are accessible to researchers. We share all code used in the manuscript on GitHub (https://github.com/peterzhukovsky/ukb_transdiagnostic).
