# Supplementary material for: Multiscale neural signatures of major depressive, anxiety, and stress-related disorders
Source: Proc Natl Acad Sci U S A. 2022 Jun 1;119(23):e2204433119. doi: 10.1073/pnas.2204433119 (PMC9191681; doi:10.1073/pnas.2204433119)
Supplement: Supplementary File [file pnas.2204433119.sapp.pdf]

Multi-scale neural signatures of major depressive, anxiety, and stress-related disorders

Peter Zhukovsky[1,2], Michael Wainberg[1], Milos Milic[1,2], Shreejoy J Tripathy[1,2,4], Benoit H Mulsant[1,2,3], Daniel Felsky[1,2,3,5 *], Aristotle N. Voineskos[1,2,3* #]

*indicates equal contribution.

[1] Campbell Family Mental Health Research Institute, Centre for Addiction and Mental Health, Toronto, ON, Canada

[2] Department of Psychiatry, University of Toronto, Toronto, ON, Canada

[3] Institute of Medical Sciences, Temerty Faculty of Medicine, University of Toronto, Toronto, ON, Canada

[4] Department of Physiology, Temerty Faculty of Medicine, University of Toronto, Toronto, ON, Canada

[5] Dalla Lana School of Public Health, University of Toronto, Toronto, ON, Canada

[#]Corresponding author: Prof Aristotle N Voineskos, Centre for Addiction and Mental Health, 250 College St, Toronto, ON M5T 1L8, Canada, Tel.: +1 (416) 535-8501

Overview

1. Methods
    1.1. Cognitive data
    1.2. Clinical data
    1.3. Mapping the UK Biobank ICA components to Yeo 7 networks
    1.4. Multiple comparison correction and permutation testing for significance
2. Results
    2.1. Clinical features and cognitive function
    2.2. Significant effects of clinical groups on cortical thickness
    2.3. Disorder similarity
    2.4. Sensitivity analyses
    2.5. Neural correlates of cognitive function in all cases
    2.6. Neural correlates of cognitive function in MDD-, ANX- MDD+ANX and STR-
    2.7. Neural correlates of cognitive function in healthy controls
    2.8. Medication information

SUPPLEMENTARY INFORMATION

METHODS

Cognitive data

Data processing. Participants with times to complete the Trailmaking test (TMT) that exceeded 4 standard deviations from the mean were excluded; participants who took less than 100 second to complete TMT alphanumeric path were excluded; TMT alphanumeric score was calculated by penalizing each error made with a 5 second penalty and adding this penalty time to the time to complete alphanumeric path.

Other UKB cognitive tests. In addition to the tests of cognitive function we included in the main analysis, there were several other cognitive tests that we decided not to include. We selected several tests that were previously shown to be impaired in major depressive disorder (MDD) or anxiety and that showed clear relevance to executive function. The tests selected were guided by our hypotheses that executive dysfunction provides a shared neurocognitive mechanism underlying MDD and anxiety.

We nevertheless present the effects of the clinical groups compared to controls on these measures of cognition after correcting for age, age^2, age*sex and sex and site (Newcastle, Cheadle, Reading) in Supplementary Table 2. While pairs matching is a popular online game (Mah-jong being one of the most famous examples of tile/card matching), it is less commonly used in cognitive neuroscience and psychiatry research although some evidence suggests that playing Mah-jong can have beneficial effects on cognitive functioning in older adults (1). We also decided against including reaction times as they lack specificity and are affected in different neurodegenerative conditions (2) as well as schizophrenia (3). However, the underlying aetiology that leads to reaction time differences is likely different across these disorders. Next, digit span is a common measure of working memory, yet we did not find reduced digit span in our case groups. Matrix completion test was not included as it provided an additional fluid intelligence measure to the already included fluid intelligence battery. Tower test is a measure of planning that was not impaired in any other disorder apart from MDD+ANX. Finally, neuroticism is a personality trait rather than a cognitive test and was not included among tests of cognitive function. Correlations between the cognitive variables are shown in Supplementary Figure S1.

Clinical Data

One of the key limitations of the UKB data is the cross-sectional nature of the dataset, whereby ICD diagnoses refer to lifetime presence of the respective psychiatric disorder. In order to illustrate the duration since the first time a diagnosis was reported, we used the age at the time of scan (Data Field 21003), year of birth (Data Field 34) and the date when a diagnosis was first reported (Data Fields 130894 for MDD, 130906 for ANX and 130910 for STR). Further, we explored the duration since the last episode based on self-reported symptoms of depression (Data Field 20434). Antidepressant prescription data

was extracted from linked electronic health records (primary care prescription records, Data-Field 42039). Age at first MDD episode was extracted from Data Field 20433 and age at most recent MDD episode was extracted from Data Field 20434. These items were self-reported during the first UKB visit and 60.0% of participants with a lifetime ICD diagnosis of MDD and 57.7% of participants with a lifetime ICD diagnosis of both MDD and ANX answered this question. Number of MDD episodes was also assessed using self-report (Data Field 20442) and only 45.1% of participants with a lifetime MDD diagnosis and only 40.1% of participants with a lifetime diagnosis of both MDD and ANX answered this question. Head motion was assessed using Data Field 25741. Genetic kinship or relatedness was assessed using Data Field 22021.

We used the following Data-Fields as an exclusion criteria for being part of the Control group: 130837, 130839, 130841, 130843, 130845, 130847, 130849, 130851, 130853, 130855, 130857, 130859, 130861, 130863, 130865, 130867, 130869, 130871, 130873, 130875, 130877, 130879, 130881, 130883, 130885, 130887, 130889, 130891, 130893, 130895, 130897, 130899, 130901, 130903, 130905, 130907, 130909, 130911, 130913, 130915, 130917, 130919, 130921, 130923, 130925, 130927, 130929, 130931, 130933, 130935, 130937, 130939, 130941, 130943, 130945, 130947, 130949, 130951, 130953, 130955, 130959, 130961, 130963, 130965, 130967, 130971, 130973, 130975, 130977, 130979, 130981, 130983, 130985, 130987, 130989, 130991, 130993, 130995, 130997, 130999, 131001, 131003, 131005, 131007, 131009, 131011, 131013, 131015, 131017, 131019, 131021, 131023, 131025, 131027, 131029, 131031, 131033, 131037, 131039, 131041, 131043, 131045, 131047, 131049, 131051, 131053, 131055, 131057, 131059, 131061, 131063, 131065, 131067, 131069, 131071, 131073, 131075, 131077, 131079, 131081, 131083, 131085, 131087, 131089, 131091, 131093, 131095, 131097, 131099, 131101, 131103, 131105, 131107, 131109, 131111, 131113, 131115, 131117, 131119, 131121, 131123, 131125, 131127.

	Gf	PAL	DSST	Pairs	RT	DS	Matrices	Tower	Neuroticism
**TMT**	**-0.39**	**-0.24**	**-0.43**	**0.25**	**0.23**	**-0.30**	**-0.39**	**-0.36**	0.01
**Gf**		**0.28**	**0.28**	**-0.17**	**-0.14**	**0.35**	**0.40**	**0.32**	**-0.04**
**PAL**			**0.22**	**-0.14**	**-0.11**	**0.19**	**0.26**	**0.19**	0.01
**DSST**				**-0.21**	**-0.25**	**0.19**	**0.33**	**0.33**	-0.02
**Pairs**					**0.13**	**-0.12**	**-0.20**	**-0.20**	0.02
**RT**						**-0.10**	**-0.17**	**-0.17**	0.01
**DS**							**0.26**	**0.20**	-0.03
**Matrices**								**0.32**	**-0.03**
**Tower**									**-0.04**

Supplementary Figure S1. Correlation structure of cognitive variables and neuroticism. For each pair of variables, rows with missing values in either variable were excluded from the correlation. TMT and Gf showed the strongest associations with other variables. TMT - trailmaking test; Gf – fluid intelligence; PAL – paired associated learning; DSST – digit-symbol substitution test; Pairs – pair matching game; RT – reaction times; DS – digit span; Matrices – matrix completion test; Tower – Tower test. Correlates significant at $P<0.001$ are shown in **bold**.

Mapping the UK Biobank ICA components to Yeo 7 networks

In order to map the UKB ICA components to the Yeo 7 networks, we obtained a volumetric parcellation of the Yeo networks from Freesurfer online resources (https://surfer.nmr.mgh.harvard.edu/fswiki/CorticalParcellation_Yeo2011). We calculated the proportion of voxels in each of the ICA components that fell in each of the 7 Yeo networks (Supplementary Table 1) and assigned each independent component (IC) to the network that encapsulated the highest proportion of voxels of that IC. Exceptions were first, IC-18 that encompassed subcortical regions, notably the striatum; second, IC-15 that encompassed the cerebellum; third IC-3 that was part of both dorsal and ventral attention networks; fourth, IC-21 that was part of the FPN, DMN and to a lesser extent VAN and finally IC-10, IC-11 and IC-12 that fell into Yeo motor and attentional networks.

ICA_d25_ID	VIS	MOT	DAN	VAN	LIM	FPN	DMN	Yeo7N
1	0.01	0.04	0.08	0.02	0.05	0.03	0.78	1.DMN
2	0.58	0.04	0.31	0.02	0.00	0.04	0.01	2.VIS
3	0.03	0.11	0.39	0.26	0.01	0.15	0.05	3.DA/VA
4	0.76	0.08	0.03	0.07	0.00	0.02	0.04	4.VIS
5	0.01	0.07	0.23	0.03	0.01	0.42	0.22	5.FPN
6	0.01	0.01	0.15	0.05	0.04	0.41	0.32	6.FPN
7	0.16	0.01	0.24	0.06	0.02	0.14	0.37	7.DMN
8	0.84	0.01	0.02	0.03	0.00	0.08	0.02	8.VIS
9	0.01	0.11	0.01	0.11	0.01	0.22	0.53	9.DMN
10	0.04	0.53	0.27	0.08	0.00	0.02	0.06	10.MOT/AN
11	0.02	0.67	0.04	0.25	0.01	0.00	0.02	11.MOT/AN
12	0.02	0.65	0.11	0.19	0.00	0.00	0.04	12.MOT/AN
13	0.00	0.08	0.16	0.11	0.05	0.14	0.46	13.DMN
14	0.05	0.13	0.01	0.15	0.04	0.14	0.48	14.DMN
15	0.71	0.03	0.03	0.18	0.02	0.00	0.03	15.CRB
16	0.00	0.01	0.09	0.20	0.01	0.40	0.29	16.FPN
17	0.09	0.38	0.06	0.19	0.03	0.02	0.22	17.MOT
18	0.02	0.36	0.14	0.39	0.00	0.07	0.02	18.STR
19	0.85	0.02	0.05	0.04	0.00	0.03	0.01	19.VIS
20	0.06	0.05	0.12	0.15	0.01	0.22	0.39	20.DMN
21	0.01	0.02	0.06	0.17	0.06	0.28	0.41	21.FP/DM

Supplementary Table 1. Proportion of voxels in each of the ICA components falling under each of the Yeo 7 networks. Initial ICA results included 25 components, but 4 of them were deemed to be representing noise by the UKB preprocessing pipeline, resulting in 21 components mapped here. FPN – frontoparietal network; DMN – default mode network; VIS – visual network; MOT – motor network; DA/VA – dorsal and ventral attentional networks; AN – attentional networks; STR – striatal network; CRB – cerebellum.

Multiple comparison correction and permutation testing for significance

Main approach. We use permutation testing a) to show which case-control differences were significant and b) to show which unthresholded case-control brain maps were significantly associated with each other. In our main analyses, we first construct general linear models, testing for the effects of diagnosis on cortical thickness and functional connectivity in separate sets of linear models. We include a number of variables such as site, sex and age as covariates. Each observation in the data matrix comes from a single participant. Next, re-run these linear models to obtain a random distribution of case-control differences using permutations of the outcome variable, i.e. either cortical thickness of one of the 360 regions or one of the 210 functional connectivities. We repeat the models with the reshuffled outcome variable 1,000 times and test for main effect of group and for case-control differences on these reshuffled cortical thickness and functional connectivity values. By re-running these randomized models, we generate random distributions of F-values testing for significance of the main effect of group. We also generate random distributions of case-control T-statistics. When testing whether

case-control T-maps are significantly correlated, we generate a ‘random’ distribution of correlations between these T-maps.

Spatial rotation ‘spin’ testing. The permutation procedure described above does not consider the spatial structure of brain maps. Therefore, we extended our analyses by using a spin test for parcellated brain maps (4), an application of the spin test for any surface map (5). Spin tests can be currently applied to spatial maps e.g. capturing cortical thickness, task activation or global connectivity. Spin tests for connectome data which involves fMRI connectivities between pairs of regions is not currently available. Therefore, spin testing was not applied to the the linear models or partial least squares models that focused on functional connectivity data. Further, functional connectivities used in this analysis involve partially overlapping brain maps derived from a group independent component analysis (6), which complicates the use of a spin test as the spatial relationships between components are not equivalent to a simple spatial map.

We first use the spin test to assess significance of the correlations between case-control brain maps of cortical thickness. Figure S2 shows the resulting p-values after permutation testing in the main analysis (A) and after spin testing (B). The pattern of significance was the same, though permutation of the outcome variable in the original linear models appears to be more stringent. We therefore report the more stringent results in the main analysis.

Hybrid permutation spin test. The original purpose of the spin test was to assess the similarity of pairs of brain maps. Therefore, all examples of the spin test toolbox test for associations of pairs of surface maps, where each observation is a region of interest. In our analyses of linear models of cortical thickness, the units of observation are individual participants. However, we have worked to combine the spin/rotation method with regular permutation testing in our cortical thickness analyses. This approach allows us to preserve the brain map structure for each subject-level brain map and still generate random distributions of linear effects of diagnosis. The original approach involved reshuffling the outcome variable for each brain region in isolation. On the other hand, in our proposed novel approach each permutation involves assigning a different rotation of the cortical thickness map for each subject. We then run linear models on cortical thickness data from each of the 360 regions. We first generated 12,214 rotations, one for each subject. During each permutation, we assign one of these rotations to each subject at random. We rotate each subject’s brain map and repeat the linear model analysis for each region. This procedure is repeated 1,000 times. P-values are then calculated by comparing the actual F-values with the randomized distributions of F-values. As before, when a significant main effect of diagnosis is found (permutation $p<0.05$), we use the T-tests from the linear model (Bonferroni corrected $p<0.0125$) to test for differences between each of the case groups vs controls. We show the significant case-control differences in Supplementary Table 4. Similar to the original analysis, we also generate p-values for the correlations assessing disorder similarity (Figure S2C).

A	ANX-	MDD+ ANX	STR-
MDD-	0	0	0.992
ANX-		0	0.745
MDD+ANX			0.881

B	ANX-	MDD+ ANX	STR-
MDD-	0	0	0.08
ANX-		0	0.07
MDD+ANX			0.17

C	ANX-	MDD+ ANX	STR-
MDD-	0.003	0	0.881
ANX-		0.001	0.675
MDD+ANX			0.765

Supplementary Figure S2. P-values for the disorder similarity correlations after regular permutation testing (A), after the spin test (B) and after the hybrid combination of regular permutation testing and spatial rotation (C) of the cortical thickness maps. Zeros indicate a p-value of $p<0.001$.

RESULTS

Clinical features and cognitive function

As shown in the Supplementary Figure S3, the first time a clinical diagnosis was reported was within 10-20 years from the time of the MRI scan for most participants. Moreover, many of the participants in the MDD- and MDD+ANX groups also showed higher PHQ-2 scores suggesting that depressive symptoms were present in these groups at the time of scan (Supplementary Figure S4, Table 1).

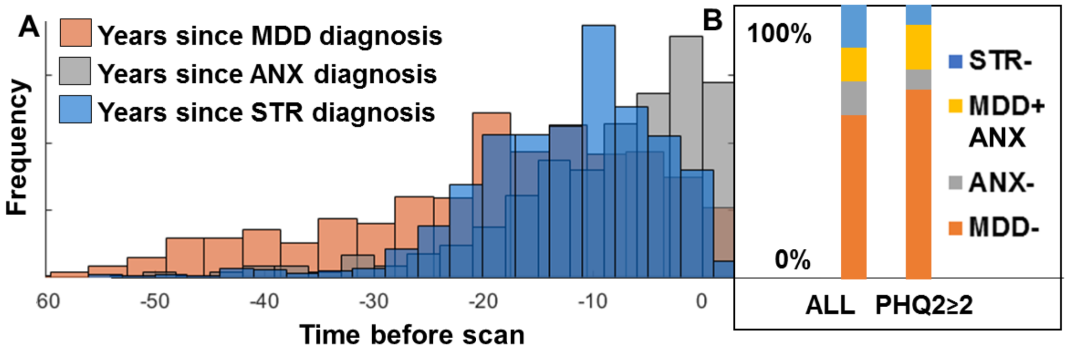

Supplementary Figure S3. Clinical sample characteristics. Histograms (A) illustrate the number of years since the first time a diagnosis of major depressive disorder (MDD, F32), a non-phobic anxiety disorder (ANX, F41) or a stressor-related disorder (STR, F43) was reported. We further broke the sample down into each of the groups shown in (B). The stacked bar chart on the left includes the proportion of all participants (with functional connectivity data) who had a lifetime diagnosis of MDD-, ANX-, MDD+ANX or STR- at the time of scan. The stacked bar chart on the right shows the proportion of participants falling into each of the clinical groups when considering only participants with a PHQ-2 score of two or higher.

Over 33% of participants with a lifetime diagnosis of MDD, ANX, or STR reported experiencing the most recent MDD episode during the last five years and over 60% of participants with a lifetime diagnosis of MDD, ANX, or STR reported such an episode in the last ten years (Supplementary Figure S4).

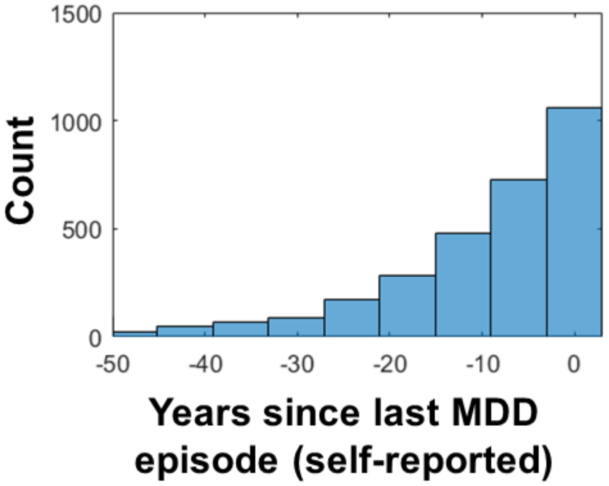

Supplementary Figure S4. Number of years since the last MDD episode based on self-report (Data Field 20434).

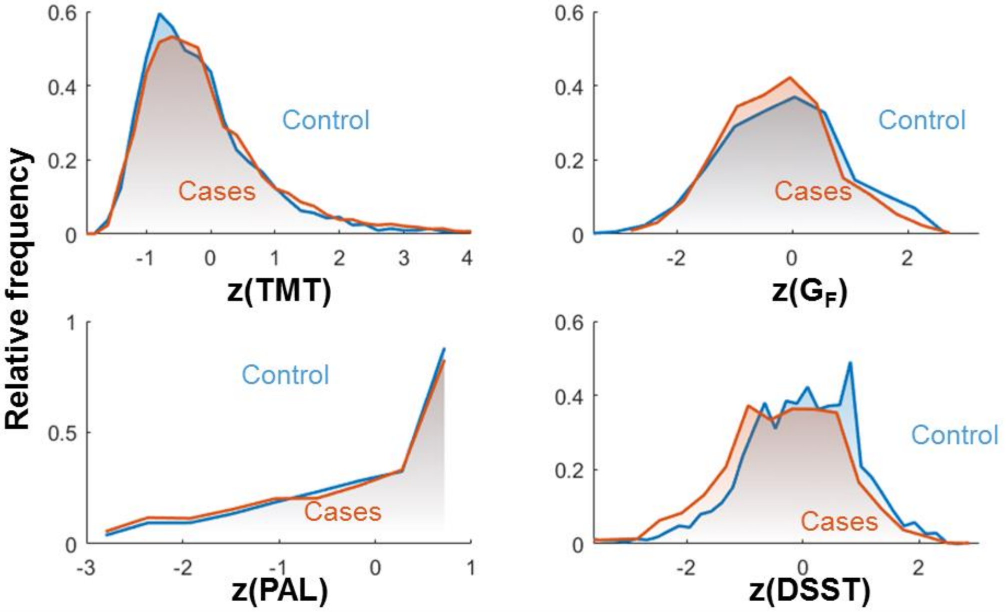

Supplementary Figure S5. Distributions of cognitive scores in control and case groups. TMT: trail making test, G_F: fluid intelligence, PAL: paired associates learning; DSST: digit-symbol substitution. Higher values on TMT indicate longer time to complete the path and thus worse performance, whereas higher values on the other tests are indicative of better performance.

Cognitive Test	Statistic	MDD-	ANX-	MDD+ANX	STR-
**TMT**	*beta*	**0.06**	**0.12**	**0.19**	0.06
	SE	**0.03**	**0.04**	**0.05**	0.04
	T-stat	**2.33**	**2.70**	**3.98**	1.33
	P-val	**0.041**	**0.019**	**3E-04**	0.236
**Gf**	*beta*	**-0.05**	**-0.14**	**-0.16**	**-0.08**
	SE	**0.02**	**0.04**	**0.04**	**0.04**
	T-stat	**-2.33**	**-3.56**	**-4.03**	**-2.29**
	P-val	**0.041**	**0.001**	**3E-04**	**0.041**
**PAL**	*beta*	-0.04	**-0.13**	-0.05	-0.06
	SE	0.03	**0.05**	0.05	0.04
	T-stat	-1.54	**-2.91**	-1.09	-1.51
	P-val	0.175	**0.011**	0.334	0.181
**DSST**	*beta*	**-0.12**	**-0.11**	**-0.28**	**-0.09**
	SE	**0.02**	**0.04**	**0.04**	**0.04**
	T-stat	**-5.20**	**-2.61**	**-6.18**	**-2.30**
	P-val	**1E-06**	**0.022**	**5E-09**	**0.041**
**Pairs**	*beta*	**0.05**	0.05	**0.22**	**0.08**
	SE	**0.02**	0.04	**0.04**	**0.04**
	T-stat	**2.59**	1.24	**5.71**	**2.17**
	P-val	**0.022**	0.267	**8E-08**	**0.053**
**RT**	*beta*	**0.06**	0.07	**0.14**	0.00
	SE	**0.02**	0.04	**0.04**	0.03
	T-stat	**2.83**	1.95	**3.55**	0.00
	P-val	**0.013**	0.086	**0.001**	0.999
**Digit Span**	*beta*	-0.05	-0.03	0.00	-0.07
	SE	0.03	0.05	0.05	0.04
	T-stat	-1.87	-0.58	0.06	-1.72
	P-val	0.098	0.643	0.979	0.126
**Matrices**	*beta*	-0.03	-0.08	-0.10	-0.01
	SE	0.03	0.05	0.05	0.04
	T-stat	-1.07	-1.86	-2.18	-0.19
	P-val	0.334	0.098	0.053	0.892
**Tower**	*beta*	-0.01	-0.01	**-0.16**	-0.06
	SE	0.03	0.05	**0.05**	0.04
	T-stat	-0.46	-0.22	**-3.35**	-1.42
	P-val	0.718	0.892	**0.003**	0.206
**Neuroticism**	*beta*	**0.79**	**0.53**	**1.04**	**0.28**
	SE	**0.02**	**0.04**	**0.04**	**0.03**
	T-stat	**37.90**	**13.89**	**27.03**	**8.04**
	P-val	**<0.001**	**<0.001**	**<0.001**	**1E-14**

Supplementary Table 2. Effects of clinical groups on cognition and neuroticism. All beta coefficients are standardized. For instance, neuroticism scores in the MDD group were 0.79 standard deviations higher than neuroticism scores in the control participants. P-values shown here were corrected using false discovery rate with the Benjamini-Hochberg method. $P_{FDR}<0.05$ are highlighted in bold.

Significant effects of diagnosis on cortical thickness

The regions of significant differences after permutation testing are shown in Supplementary Table 3. In order to correct for multiple comparisons with the case-control differences, we used the following thresholds: $P_{PERMUTATON}<0.05$ to assess whether the overall group effect was significant and in addition, we Bonferroni-corrected (P<0.0125) the post-hoc case-control comparisons of MDD-, ANX-, STR- and MDD+ANX vs control group.

Supplementary Table 3A. Significant effects of MDD on cortical thickness

HCP Region	HOA region	T-statistic	P-Value (Unc.)	P-Value (Bonf.)	Effect Size
R_a32pr_ROI	Anterior Cingulate	-3.81	<0.001	0.001	-0.069
R_p24pr_ROI	Anterior Cingulate Cortex	-2.66	0.008	0.031	-0.048
L_p32pr_ROI	Anterior Cingulate Cortex (anterior)	-4.00	<0.001	<0.001	-0.072
L_10v_ROI	Frontal Medial Cortex	-2.62	0.009	0.035	-0.047
R_10v_ROI	Frontal Medial Cortex	-3.07	0.002	0.008	-0.056
L_FOP4_ROI	Frontal Operculum Cortex	-2.74	0.006	0.025	-0.050
R_8BL_ROI	Frontal Pole	-2.52	0.012	0.048	-0.046
L_10d_ROI	Frontal Pole (anterior)	-2.57	0.010	0.041	-0.046
L_9a_ROI	Frontal Pole (dorsal anterior)	-3.29	0.001	0.004	-0.060
R_p10p_ROI	Frontal Pole (medial)	-2.52	0.012	0.047	-0.046
R_A1_ROI	Heschls Gyrus	2.52	0.012	0.047	0.046
L_6r_ROI	Inferior Frontal Gyrus, pars opercularis	-2.96	0.003	0.012	-0.054
L_IFSa_ROI	Inferior Frontal Gyrus, pars triangularis	-2.83	0.005	0.019	-0.051
L_IFSp_ROI	Inferior Frontal Gyrus/Middle Frontal Gyrus	-3.16	0.002	0.006	-0.057
R_MI_ROI	Insular Cortex (dorsal)	-2.88	0.004	0.016	-0.052
R_FOP2_ROI	Insular Cortex (dorsal)	-3.00	0.003	0.011	-0.054
L_Ig_ROI	Insular Cortex/Parietal Operculum	-2.83	0.005	0.019	-0.051
L_TPOJ2_ROI	Lateral Occipital Cortex, inferior division	-2.79	0.005	0.021	-0.051
L_24dd_ROI	Medial Postcentral Gyrus	-3.04	0.002	0.010	-0.055
L_8BM_ROI	Medial Superior Frontal Gyrus	-3.51	<0.001	0.002	-0.063
R_8BM_ROI	Medial Superior Frontal Gyrus	-3.05	0.002	0.009	-0.055
L_46_ROI	Middle Frontal Gyrus	-2.66	0.008	0.031	-0.048
R_8Av_ROI	Middle Frontal Gyrus	-3.55	<0.001	0.002	-0.064
R_46_ROI	Middle Frontal Gyrus	-2.67	0.008	0.030	-0.048
L_TE1a_ROI	Middle Temporal Gyrus (anterior)	-3.43	0.001	0.002	-0.062
R_V1_ROI	Occipital Cortex	2.90	0.004	0.015	0.053
L_d32_ROI	Paracingulate Gyrus	-3.96	<0.001	<0.001	-0.072
R_d32_ROI	Paracingulate Gyrus	-4.77	<0.001	<0.001	-0.086
L_PeEc_ROI	Parahippocampal Gyrus	-3.06	0.002	0.009	-0.055
L_RI_ROI	Parietal Opercular Cortex	-3.44	0.001	0.002	-0.062
R_PFcm_ROI	Parietal Opercular Cortex	-2.51	0.012	0.048	-0.046
L_6mp_ROI	Precentral Gyrus	-2.56	0.010	0.041	-0.046
R_FEF_ROI	Precentral Gyrus	-2.93	0.003	0.014	-0.053
L_SFL_ROI	Superior Frontal Gyrus	-2.75	0.006	0.024	-0.050
L_6ma_ROI	Superior Frontal Gyrus	-3.17	0.002	0.006	-0.057
R_SFL_ROI	Superior Frontal Gyrus	-3.92	<0.001	<0.001	-0.071
R_6ma_ROI	Superior Frontal Gyrus	-2.76	0.006	0.023	-0.050
R_9m_ROI	Superior Frontal Gyrus/Frontal Pole	-3.16	0.002	0.006	-0.057

L_SCEF_ROI	Supplementary Motor Cortex	-4.02	<0.001	<0.001	-0.073
R_SCEF_ROI	Supplementary Motor Cortex	-3.15	0.002	0.006	-0.057
L_PFt_ROI	Supramarginal Gyrus (anterior)	-3.15	0.002	0.006	-0.057
L_TGd_ROI	Temporal Pole	-3.16	0.002	0.006	-0.057
L_STGa_ROI	Temporal Pole (aTL)	-3.05	0.002	0.009	-0.055

Supplementary Table 3B. Significant effects of comorbid MDD and anxiety on cortical thickness

**HCP Region**	**HOA region**	**T-statistic**	**P-Value (Unc.)**	**P-Value (Bonf.)**	**Effect Size**
R_PFm_ROI	Angular Gyrus/Supramarginal Gyrus	-2.99	0.003	0.011	-0.054
L_a32pr_ROI	Anterior Cingulate	-3.47	0.001	0.002	-0.063
R_a32pr_ROI	Anterior Cingulate	-3.18	0.001	0.006	-0.058
R_p24_ROI	Anterior Cingulate	-4.81	<0.001	<0.001	-0.087
R_p24pr_ROI	Anterior Cingulate Cortex	-2.97	0.003	0.012	-0.054
L_p32pr_ROI	Anterior Cingulate Cortex (anterior)	-4.29	<0.001	<0.001	-0.078
R_a24pr_ROI	Anterior Cingulate Cortex (anterior)	-2.73	0.006	0.026	-0.049
R_p32pr_ROI	Anterior Cingulate Cortex (anterior)	-3.81	<0.001	0.001	-0.069
R_10v_ROI	Frontal Medial Cortex	-3.31	0.001	0.004	-0.060
R_47s_ROI	Frontal Orbital Cortex (Medial)	-2.89	0.004	0.016	-0.052
L_9-46d_ROI	Frontal Pole	-2.50	0.012	0.050	-0.045
R_9-46d_ROI	Frontal Pole	-3.22	0.001	0.005	-0.058
R_9p_ROI	Frontal Pole (anterior)	-3.03	0.002	0.010	-0.055
L_p10p_ROI	Frontal Pole (medial)	-2.77	0.006	0.022	-0.050
R_p10p_ROI	Frontal Pole (medial)	-2.94	0.003	0.013	-0.053
L_52_ROI	Heschls Gyrus	-2.90	0.004	0.015	-0.052
L_6r_ROI	Inferior Frontal Gyrus, pars opercularis	-3.05	0.002	0.009	-0.055
R_6r_ROI	Inferior Frontal Gyrus, pars opercularis	-3.04	0.002	0.010	-0.055
R_TGv_ROI	Inferior Temporal Gyrus, anterior division	-2.96	0.003	0.012	-0.054
L_PoI1_ROI	Insular Cortex	-4.03	<0.001	<0.001	-0.073
R_PoI1_ROI	Insular Cortex	-3.12	0.002	0.007	-0.056
L_AAIC_ROI	Insular Cortex (anterior)	-2.64	0.008	0.033	-0.048
R_MI_ROI	Insular Cortex (dorsal)	-2.58	0.010	0.040	-0.047
L_PoI2_ROI	Insular Cortex (posterior)	-3.28	0.001	0.004	-0.059
R_PoI2_ROI	Insular Cortex (posterior)	-3.37	0.001	0.003	-0.061
L_Ig_ROI	Insular Cortex/Parietal Operculum	-3.07	0.002	0.009	-0.055
R_Ig_ROI	Insular Cortex/Parietal Operculum	-2.53	0.011	0.046	-0.046
L_OP2-3_ROI	Insular/Parietal Opercular Cortex	-2.86	0.004	0.017	-0.052
L_V6A_ROI	Lateral Occipital Cortex	-2.83	0.005	0.019	-0.051
L_TPOJ2_ROI	Lateral Occipital Cortex, inferior division	-3.29	0.001	0.004	-0.059
L_24dd_ROI	Medial Postcentral Gyrus	-3.05	0.002	0.009	-0.055
L_24dv_ROI	Medial Postcentral Gyrus	-3.63	<0.001	0.001	-0.066
R_8BM_ROI	Medial Superior Frontal Gyrus	-2.56	0.011	0.043	-0.046
R_8Av_ROI	Middle Frontal Gyrus	-2.67	0.007	0.030	-0.048
R_46_ROI	Middle Frontal Gyrus	-2.83	0.005	0.019	-0.051
L_TE1a_ROI	Middle Temporal Gyrus (anterior)	-3.26	0.001	0.004	-0.059
R_TE1a_ROI	Middle Temporal Gyrus (anterior)	-2.74	0.006	0.025	-0.050
R_STSvp_ROI	Middle Temporal Gyrus (posterior)	-2.94	0.003	0.013	-0.053
R_TPOJ1_ROI	Middle Temporal Gyrus, temporooccipital part	-2.86	0.004	0.017	-0.052
L_d32_ROI	Paracingulate Gyrus	-3.31	0.001	0.004	-0.060
R_d32_ROI	Paracingulate Gyrus	-2.59	0.010	0.039	-0.047

L_H_ROI	Parahippocampal Gyrus	-3.65	<0.001	0.001	-0.066
R_H_ROI	Parahippocampal Gyrus	-4.19	<0.001	<0.001	-0.076
R_PeEc_ROI	Parahippocampal Gyrus	-3.66	<0.001	0.001	-0.066
L_OP1_ROI	Parietal Opercular Cortex	-2.63	0.008	0.034	-0.048
L_RI_ROI	Parietal Opercular Cortex	-3.05	0.002	0.009	-0.055
L_PFcm_ROI	Parietal Opercular Cortex	-3.03	0.002	0.010	-0.055
R_RI_ROI	Parietal Opercular Cortex	-4.21	<0.001	<0.001	-0.076
R_PFcm_ROI	Parietal Opercular Cortex	-2.65	0.008	0.033	-0.048
R_PBelt_ROI	Planum Temporale (STG)	-3.05	0.002	0.009	-0.055
R_LBelt_ROI	Planum Temporale (STG)	-2.93	0.003	0.014	-0.053
L_PFop_ROI	Postcentral Gyrus (ventral)	-2.63	0.009	0.034	-0.048
R_23d_ROI	Posterior Cingulate Cortex	-2.84	0.005	0.018	-0.051
R_ProS_ROI	Posterior Cingulate Cortex (ventral)	-3.72	<0.001	0.001	-0.067
L_6mp_ROI	Precentral Gyrus	-2.63	0.009	0.035	-0.048
R_FEF_ROI	Precentral Gyrus	-2.77	0.006	0.022	-0.050
R_6a_ROI	Precentral Gyrus (medial)	-2.89	0.004	0.016	-0.052
L_7m_ROI	Precuneous	-2.81	0.005	0.020	-0.051
R_25_ROI	Subcallosal Cortex (Subgenual ACC)	-3.98	<0.001	<0.001	-0.072
L_SFL_ROI	Superior Frontal Gyrus	-2.69	0.007	0.029	-0.049
L_6ma_ROI	Superior Frontal Gyrus	-3.21	0.001	0.005	-0.058
R_SFL_ROI	Superior Frontal Gyrus	-3.45	0.001	0.002	-0.063
R_9m_ROI	Superior Frontal Gyrus/Frontal Pole	-3.58	<0.001	0.001	-0.065
L_SCEF_ROI	Supplementary Motor Cortex	-3.08	0.002	0.008	-0.056
L_STV_ROI	Supramarginal Gyrus (posterior)/Angular Gyrus	-4.09	<0.001	<0.001	-0.074
L_PF_ROI	Supramarginal Gyrus, anterior division	-2.62	0.009	0.035	-0.047
R_IP2_ROI	Supramarginal Gyrus, posterior division	-3.36	0.001	0.003	-0.061
R_PHA2_ROI	Temporal Fusiform Cortex, posterior division	-2.72	0.006	0.026	-0.049
R_TGd_ROI	Temporal Pole	-3.19	0.001	0.006	-0.058

Supplementary Table 3C. Significant effects of anxiety on cortical thickness

HCP Region	HOA region	T-statistic	P-Value (Unc.)	P-Value (Bonf.)	Effect Size
L_STSvp_ROI	Middle Temporal Gyrus (posterior)	-2.61	0.009	0.036	-0.047
L_PFcm_ROI	Parietal Opercular Cortex	-2.57	0.010	0.040	-0.047
L_STV_ROI	Supramarginal Gyrus (posterior)/Angular Gyrus	-3.91	<0.001	<0.001	-0.071
L_TGd_ROI	Temporal Pole	-2.57	0.010	0.041	-0.046

Supplementary Table 3D. Significant effects of stressor-related disorders on cortical thickness

HCP Region	HOA region	T-statistic	P-Value (Unc.)	P-Value (Bonf.)	Effect Size
L_VMV1_ROI	Lingual Gyrus	-3.09	0.002	0.008	-0.056
L_PHA1_ROI	Parahippocampal/Lingual Gyrus	-2.51	0.012	0.049	-0.045

We further present the results of the hybrid permutation analysis in Supplementary Table 4. The regular and the hybrid permutation approaches produced highly consistent results, although the hybrid permutation analysis returned fewer significantly different regions compared to the regular permutation test. The same healthy control group was used for the regular and the hybrid permutation analysis. The healthy control group was picked at random to match the cases in sample size, age and sex.

Supplementary Table 4A. Significant effects of MDD on cortical thickness

HCP Region	HOA region	T-statistic	P-Value (Unc.)	P-Value (Bonf.)	Effect Size
R_a32pr_ROI	Anterior Cingulate	-3.81	<0.001	0.001	-0.069
R_p24pr_ROI	Anterior Cingulate Cortex	-2.66	0.008	0.031	-0.048
L_p32pr_ROI	Anterior Cingulate Cortex (anterior)	-4.00	<0.001	<0.001	-0.072
R_10v_ROI	Frontal Medial Cortex	-3.07	0.002	0.008	-0.056
R_8BL_ROI	Frontal Pole	-2.52	0.012	0.048	-0.046
L_10d_ROI	Frontal Pole (anterior)	-2.57	0.010	0.041	-0.046
L_9a_ROI	Frontal Pole (dorsal anterior)	-3.29	0.001	0.004	-0.060
R_p10p_ROI	Frontal Pole (medial)	-2.52	0.012	0.047	-0.046
R_A1_ROI	Heschls Gyrus	2.52	0.012	0.047	0.046
L_6r_ROI	Inferior Frontal Gyrus, pars opercularis	-2.96	0.003	0.012	-0.054
L_IFSp_ROI	Inferior Frontal Gyrus/Middle Frontal Gyrus	-3.16	0.002	0.006	-0.057
R_MI_ROI	Insular Cortex (dorsal)	-2.88	0.004	0.016	-0.052
L_Ig_ROI	Insular Cortex/Parietal Operculum	-2.83	0.005	0.019	-0.051
L_TPOJ2_ROI	Lateral Occipital Cortex, inferior division	-2.79	0.005	0.021	-0.051
L_24dd_ROI	Medial Postcentral Gyrus	-3.04	0.002	0.010	-0.055
L_8BM_ROI	Medial Superior Frontal Gyrus	-3.51	<0.001	0.002	-0.063
R_8BM_ROI	Medial Superior Frontal Gyrus	-3.05	0.002	0.009	-0.055
L_46_ROI	Middle Frontal Gyrus	-2.66	0.008	0.031	-0.048
R_8Av_ROI	Middle Frontal Gyrus	-3.55	<0.001	0.002	-0.064
R_46_ROI	Middle Frontal Gyrus	-2.67	0.008	0.030	-0.048
L_TE1a_ROI	Middle Temporal Gyrus (anterior)	-3.43	0.001	0.002	-0.062
R_V1_ROI	Occipital Cortex	2.90	0.004	0.015	0.053
L_d32_ROI	Paracingulate Gyrus	-3.96	<0.001	<0.001	-0.072
R_d32_ROI	Paracingulate Gyrus	-4.77	<0.001	<0.001	-0.086
L_RI_ROI	Parietal Opercular Cortex	-3.44	0.001	0.002	-0.062
R_PFcm_ROI	Parietal Opercular Cortex	-2.51	0.012	0.048	-0.046
R_FEF_ROI	Precentral Gyrus	-2.93	0.003	0.014	-0.053
L_SFL_ROI	Superior Frontal Gyrus	-2.75	0.006	0.024	-0.050
L_6ma_ROI	Superior Frontal Gyrus	-3.17	0.002	0.006	-0.057
R_SFL_ROI	Superior Frontal Gyrus	-3.92	<0.001	<0.001	-0.071
R_6ma_ROI	Superior Frontal Gyrus	-2.76	0.006	0.023	-0.050
R_9m_ROI	Superior Frontal Gyrus/Frontal Pole	-3.16	0.002	0.006	-0.057
L_SCEF_ROI	Supplementary Motor Cortex	-4.02	<0.001	<0.001	-0.073
R_SCEF_ROI	Supplementary Motor Cortex	-3.15	0.002	0.006	-0.057
L_TGd_ROI	Temporal Pole	-3.16	0.002	0.006	-0.057
L_STGa_ROI	Temporal Pole (aTL)	-3.05	0.002	0.009	-0.055

Supplementary Table 4B. Significant effects of comorbid MDD and anxiety on cortical thickness

HCP Region	HOA region	T-statistic	P-Value (Unc.)	P-Value (Bonf.)	Effect Size
R_PFm_ROI	Angular Gyrus/Supramarginal Gyrus	-2.99	0.003	0.011	-0.054
L_a32pr_ROI	Anterior Cingulate	-3.47	0.001	0.002	-0.063
R_a32pr_ROI	Anterior Cingulate	-3.18	0.001	0.006	-0.058
R_p24_ROI	Anterior Cingulate	-4.81	<0.001	<0.001	-0.087
R_p24pr_ROI	Anterior Cingulate Cortex	-2.97	0.003	0.012	-0.054
L_p32pr_ROI	Anterior Cingulate Cortex (anterior)	-4.29	<0.001	<0.001	-0.078
R_p32pr_ROI	Anterior Cingulate Cortex (anterior)	-3.81	<0.001	0.001	-0.069
R_10v_ROI	Frontal Medial Cortex	-3.31	0.001	0.004	-0.060
R_47s_ROI	Frontal Orbital Cortex (Medial)	-2.89	0.004	0.016	-0.052
R_9-46d_ROI	Frontal Pole	-3.22	0.001	0.005	-0.058
R_9p_ROI	Frontal Pole (anterior)	-3.03	0.002	0.010	-0.055
L_p10p_ROI	Frontal Pole (medial)	-2.77	0.006	0.022	-0.050
R_p10p_ROI	Frontal Pole (medial)	-2.94	0.003	0.013	-0.053
L_6r_ROI	Inferior Frontal Gyrus, pars opercularis	-3.05	0.002	0.009	-0.055
R_6r_ROI	Inferior Frontal Gyrus, pars opercularis	-3.04	0.002	0.010	-0.055
R_TGv_ROI	Inferior Temporal Gyrus, anterior division	-2.96	0.003	0.012	-0.054
L_PoI1_ROI	Insular Cortex	-4.03	<0.001	<0.001	-0.073
L_AAIC_ROI	Insular Cortex (anterior)	-2.64	0.008	0.033	-0.048
R_MI_ROI	Insular Cortex (dorsal)	-2.58	0.010	0.040	-0.047
L_PoI2_ROI	Insular Cortex (posterior)	-3.28	0.001	0.004	-0.059
R_PoI2_ROI	Insular Cortex (posterior)	-3.37	0.001	0.003	-0.061
L_Ig_ROI	Insular Cortex/Parietal Operculum	-3.07	0.002	0.009	-0.055
L_TPOJ2_ROI	Lateral Occipital Cortex, inferior division	-3.29	0.001	0.004	-0.059
L_24dd_ROI	Medial Postcentral Gyrus	-3.05	0.002	0.009	-0.055
L_24dv_ROI	Medial Postcentral Gyrus	-3.63	<0.001	0.001	-0.066
R_8BM_ROI	Medial Superior Frontal Gyrus	-2.56	0.011	0.043	-0.046
R_8Av_ROI	Middle Frontal Gyrus	-2.67	0.007	0.030	-0.048
R_46_ROI	Middle Frontal Gyrus	-2.83	0.005	0.019	-0.051
L_TE1a_ROI	Middle Temporal Gyrus (anterior)	-3.26	0.001	0.004	-0.059
R_STSvp_ROI	Middle Temporal Gyrus (posterior)	-2.94	0.003	0.013	-0.053
L_d32_ROI	Paracingulate Gyrus	-3.31	0.001	0.004	-0.060
R_d32_ROI	Paracingulate Gyrus	-2.59	0.010	0.039	-0.047
L_H_ROI	Parahippocampal Gyrus	-3.65	<0.001	0.001	-0.066
R_H_ROI	Parahippocampal Gyrus	-4.19	<0.001	<0.001	-0.076
R_PeEc_ROI	Parahippocampal Gyrus	-3.66	<0.001	0.001	-0.066
L_OP1_ROI	Parietal Opercular Cortex	-2.63	0.008	0.034	-0.048
L_RI_ROI	Parietal Opercular Cortex	-3.05	0.002	0.009	-0.055
L_PFcm_ROI	Parietal Opercular Cortex	-3.03	0.002	0.010	-0.055
R_RI_ROI	Parietal Opercular Cortex	-4.21	<0.001	<0.001	-0.076
R_PFcm_ROI	Parietal Opercular Cortex	-2.65	0.008	0.033	-0.048
R_PBelt_ROI	Planum Temporale (STG)	-3.05	0.002	0.009	-0.055
R_23d_ROI	Posterior Cingulate Cortex	-2.84	0.005	0.018	-0.051
R_ProS_ROI	Posterior Cingulate Cortex (ventral)	-3.72	<0.001	0.001	-0.067
R_FEF_ROI	Precentral Gyrus	-2.77	0.006	0.022	-0.050

R_25_ROI	Subcallosal Cortex (Subgenual ACC)	-3.98	<0.001	<0.001	-0.072
L_SFL_ROI	Superior Frontal Gyrus	-2.69	0.007	0.029	-0.049
L_6ma_ROI	Superior Frontal Gyrus	-3.21	0.001	0.005	-0.058
R_SFL_ROI	Superior Frontal Gyrus	-3.45	0.001	0.002	-0.063
R_9m_ROI	Superior Frontal Gyrus/Frontal Pole	-3.58	<0.001	0.001	-0.065
L_SCEF_ROI	Supplementary Motor Cortex	-3.08	0.002	0.008	-0.056
L_STV_ROI	Supramarginal Gyrus (posterior)/Angular Gyrus	-4.09	<0.001	<0.001	-0.074
R_PHA2_ROI	Temporal Fusiform Cortex, posterior division	-2.72	0.006	0.026	-0.049
R_TGd_ROI	Temporal Pole	-3.19	0.001	0.006	-0.058

Supplementary Table 4C. Significant effects of anxiety on cortical thickness

HCP Region	HOA region	T-statistic	P-Value (Unc.)	P-Value (Bonf.)	Effect Size
L_STSvp_ROI	Middle Temporal Gyrus (posterior)	-2.61	0.009	0.036	-0.047
L_PFcm_ROI	Parietal Opercular Cortex	-2.57	0.010	0.040	-0.047
L_STV_ROI	Supramarginal Gyrus (posterior)/Angular Gyrus	-3.91	<0.001	<0.001	-0.071
L_TGd_ROI	Temporal Pole	-2.57	0.010	0.041	-0.046

Supplementary Table 4D. Significant effects of stressor-related disorders on cortical thickness

HCP Region	HOA region	T-statistic	P-Value (Unc.)	P-Value (Bonf.)	Effect Size
L_VMV1_ROI	Lingual Gyrus	-3.09	0.002	0.008	-0.056

Disorder Similarity

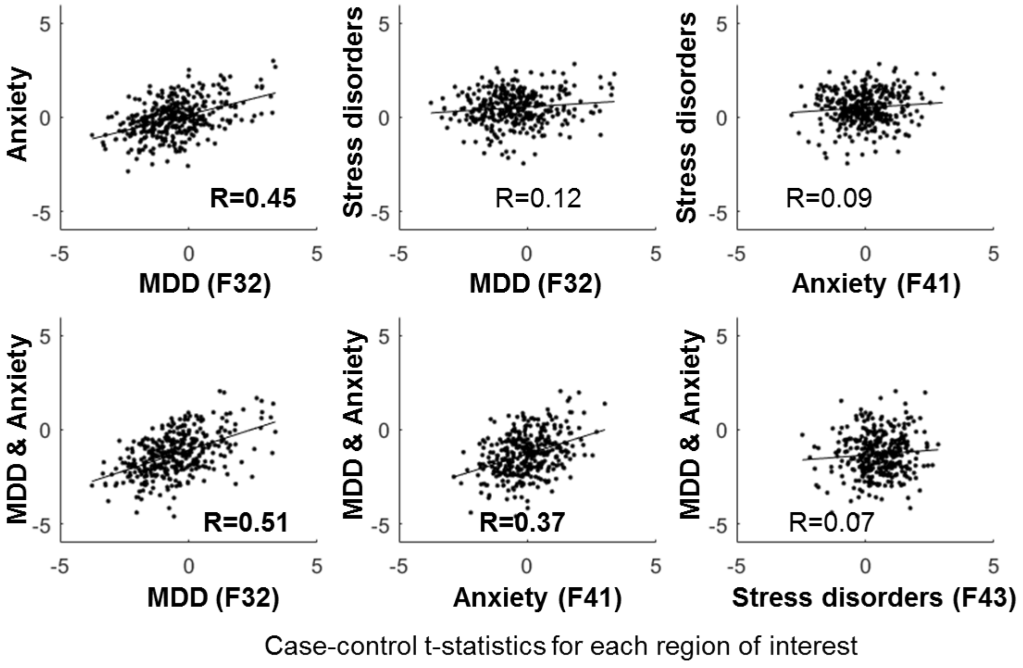

Supplementary Figure S6. Disorder similarity assessed using cortical thickness. For each pair of disorders, we show scatterplots of case-control t-statistics comparing cortical thickness. Each datapoint represents a region of interest in the 360-region HCP parcellation. Significant correlations are highlighted in **bold**.

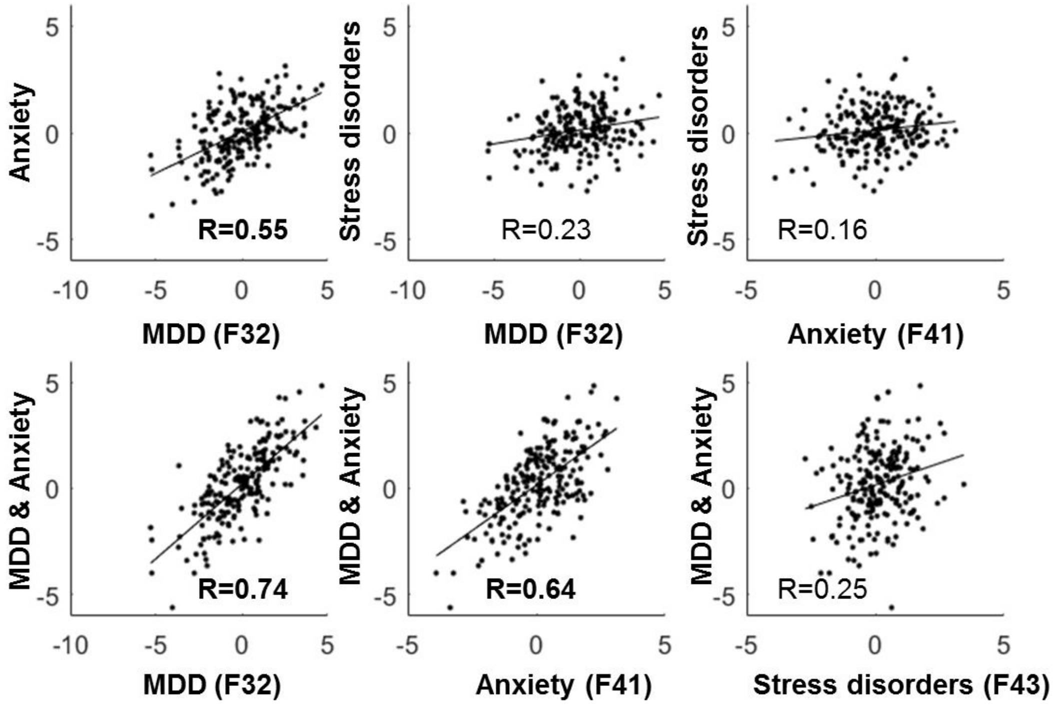

Supplementary Figure S7. Disorder similarity assessed using functional connectivity. For each pair of disorders, we show scatterplots of case-control t-statistics comparing functional connectivity. Each datapoint represents one of the 210 unique functional connectivities. Significant correlations are highlighted in **bold**.

Sensitivity analyses

We include two sensitivity analyses: firstly, we show that restricting the MDD group to active MDD only did not impact our disorder similarity results (Figure S8). We defined active MDD at the time of scanning and cognitive testing using the PHQ-2 with a cut-off score of two or greater (7).

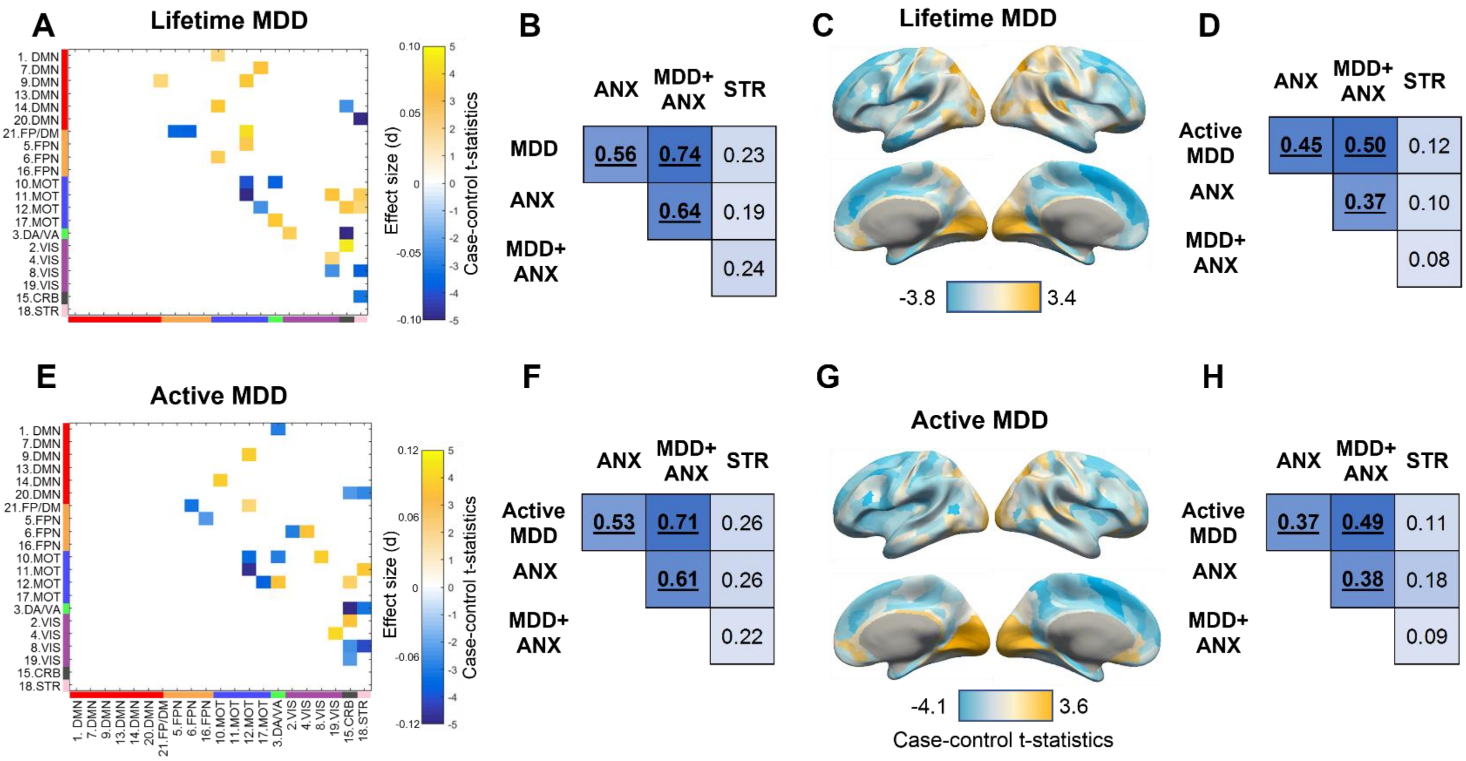

Supplementary Figure S8. Case-control differences in functional connectivity (A) and cortical thickness (C) between lifetime MDD- and healthy control groups (also shown in Figure 1 and 2) were highly consistent with the case-control differences between active MDD and controls (E, G). Focusing only on those with lifetime MDD diagnosis and PHQ2≥2 resulted in lower number of participants with active MDD (n=977 for functional connectivity and n=1,103 for cortical thickness). Disorder similarity was also highly consistent between lifetime (B) and active MDD (F).

Secondly, we repeat the case-control comparisons focusing only on unmedicated participants and show that the results were highly consistent after excluding unmedicated participants (Supplementary Figure S9).

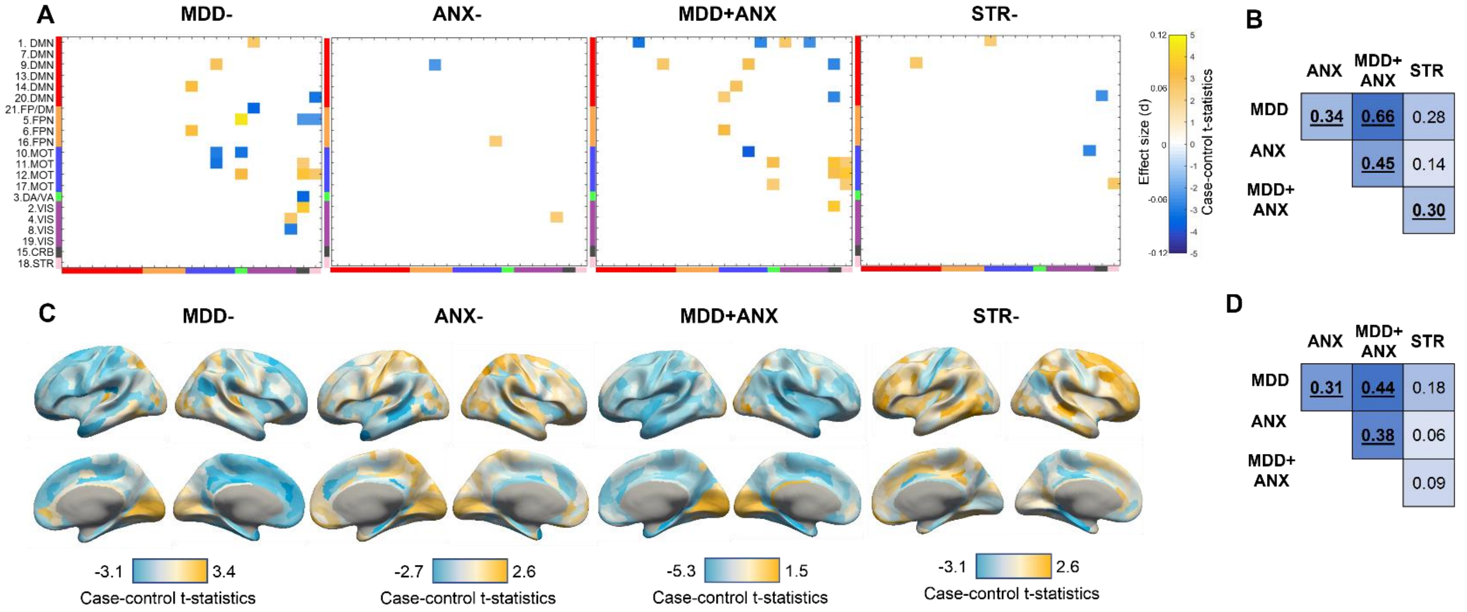

Supplementary Figure S9. Case-control comparisons for functional connectivity (A) and cortical thickness (C) after exclusion of participants taking medications were similar to the case-control maps for all participants although the sample size was substantially reduced (total n=7,646 for each analysis). Disorder similarity (B,D) was also largely consistent between the main results and this sensitivity analysis.

Neural correlates of cognitive function in all cases

In addition to presenting the correlation matrices showing the relationships between cognitive tests (TMT, Gf, PAL, and DSST) with PLS latent variable scores, we also show scatterplots of these relationships in Supplementary Figure S10. We also provide more details on PLS2 weights in Supplementary Figure S10B.

No significant brain-cognition associations between cortical thickness (instead of functional connectivity) and cognitive function were found in all cases (n=3,372, permutation p=0.185).

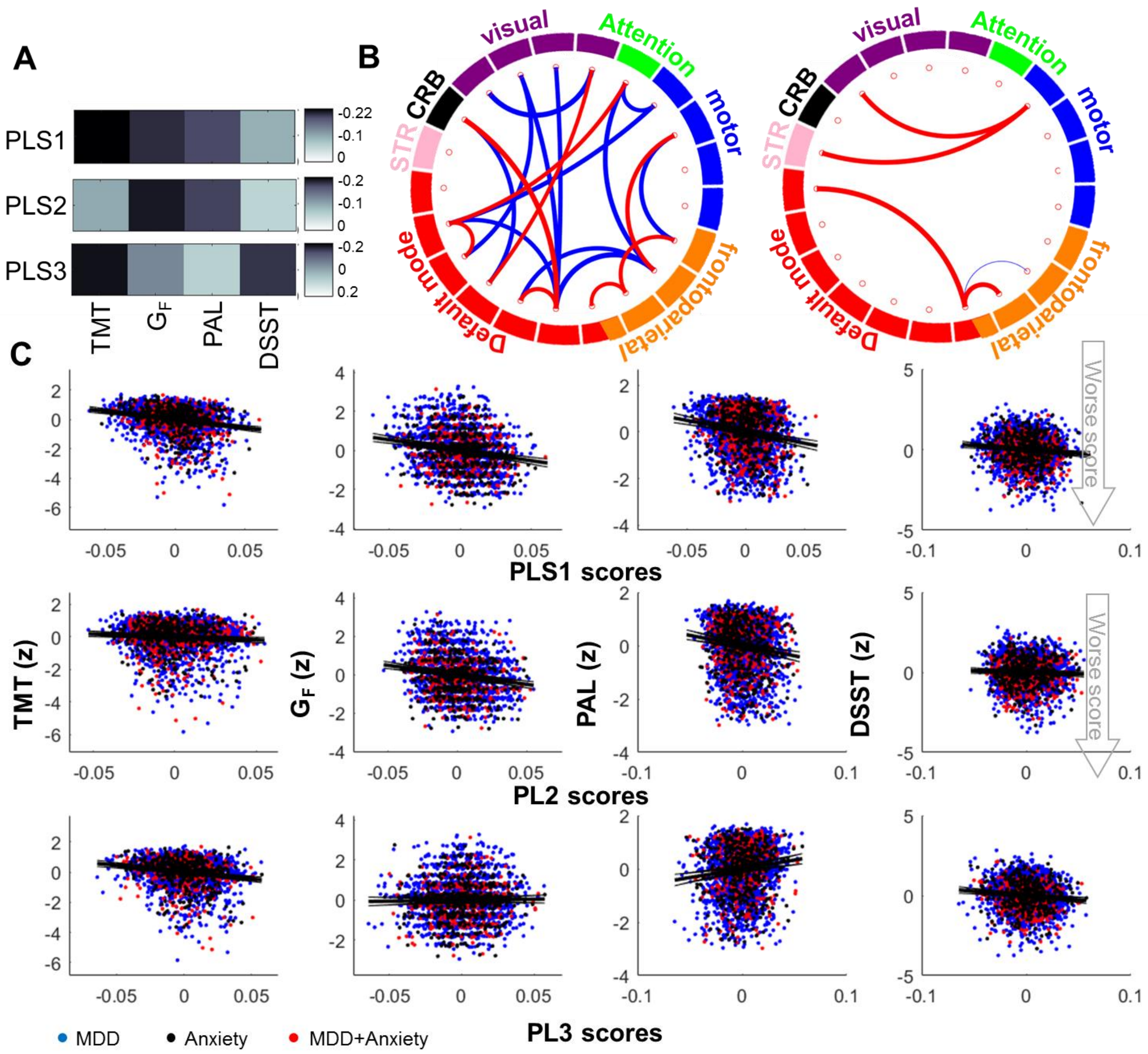

Supplementary Figure S10. (A) Associations between PLS scores and cognitive function tests in all cases. (C) In addition to scatterplots, lines of best fit and 99% confidence intervals (obtained using MATLAB R2016a, *fitlm* and *predict* functions) are shown. (B) Thresholded PLS2 weights implicated connectivities between independent components corresponding to the default mode (DMN), frontoparietal (FPN) and striatal (STR) networks. Blue connections between network components suggest that higher connectivity of those components was associated with worse fluid intelligence and paired associates learning. Red connections between network components suggest that higher connectivity of those components predicted better fluid intelligence and paired associates learning. No functional connectivities showed robust loadings on PLS3 ($|Z|<3$) and thus no graph is shown for PLS3. Network labels include: MOT – motor; CRB – cerebellum; VIS – visual; DA/VA – dorsal/ventral attention. TMT – Trailmaking path; Gf – fluid intelligence test; PAL – paired associates learning; DSST – digit-symbol substitution test.

Neural correlates of cognitive function in MDD-, ANX- MDD+ANX and STR-

A separate PLS regression found three latent variables that together explained a significant amount of variance ($P_{PERMUTATION}$<0.001) in the cognitive scores in MDD- (n=1,895). PLS1, PLS2 and PLS3 explained 5.2%, 0.8% and 1.6% of variance in cognitive scores. PLS1 scores were associated with longer times to complete trailmaking and worse scores on fluid intelligence, digit-symbol substitution and paired associate learning. Since no PLS2 or PLS3 weights showed reliable weights (|Z|<3) after bootstrapping, we did not focus on these PLS latent variables.

A PLS regression in ANX- (n=426) identified three latent variables that together explained a significant amount of variance in the cognitive scores ($P_{PERMUTATION}$=0.009). PLS1, PLS2 and PLS3 explained 10.0%, 6.9% and 6.3% of variance in cognitive scores. Similar to the other PLS analyses, PLS1 scores were associated with longer times to complete Trailmaking and worse scores on fluid intelligence, digit-symbol substitution and paired associate learning. No significant brain-cognition relationships were found in MDD+ANX or STR- (P_{PERM}>0.1).

Neural correlates of cognitive function in healthy controls

We aimed to uncover the neural correlates of worse executive function and verbal memory of the Control group (n=14,199) aiming to test whether the neural correlates of executive function is similar to those of the clinical groups presented in Figure 3. These results are shown in Supplementary Figure S11 and S12. PLS regression identified three components that explained 2.0%, 1.2% and 0.4% of variance in the four cognitive function tests (TMT, G_F, PAL and DSST). Permutation testing showed that these components together explained a significant amount of variance in executive function (P_{PERM}<0.001). The first component, PLS1, captured the most variance in the outcome variables (Supplementary Figure S12) and was associated with worse performance on each of the tests. The second component, PLS2, was associated with better performance on each of the tests.

We found 20 connectivities with normalized PLS1 weights with Z>4 and 11 connectivities with normalized PLS1 weights with Z<-4 (Figure S11B). There were 14 PLS2 weights with Z>4 and 19 PLS2 weights with Z<-4. Functional correlates of worse executive and memory performance were similar to the functional correlates of worse performance in the clinical groups, although more functional connectivity correlates were identified in the bigger sample of healthy controls.

A separate PLS model testing for brain-cognition associations with cortical thickness instead of functional connectivity was significant in controls (n=14,885, permutation p<0.001). However, it explained less variance than the functional connectivity PLS. In the cortical thickness PLS, the three components explained 0.69%, 0.37% and 0.19% of variance in the congitive function tests. The cortical thickness PLS1 scores were moderately correlated with TMT, G_F, PAL and DSST (r=-0.09. r=-0.12, r=-0.05 and r=-0.05, respectively). Given that the focus of our analyses was on the brain-cognition associations in mood disorders, we report these results in the Supplementary Information.

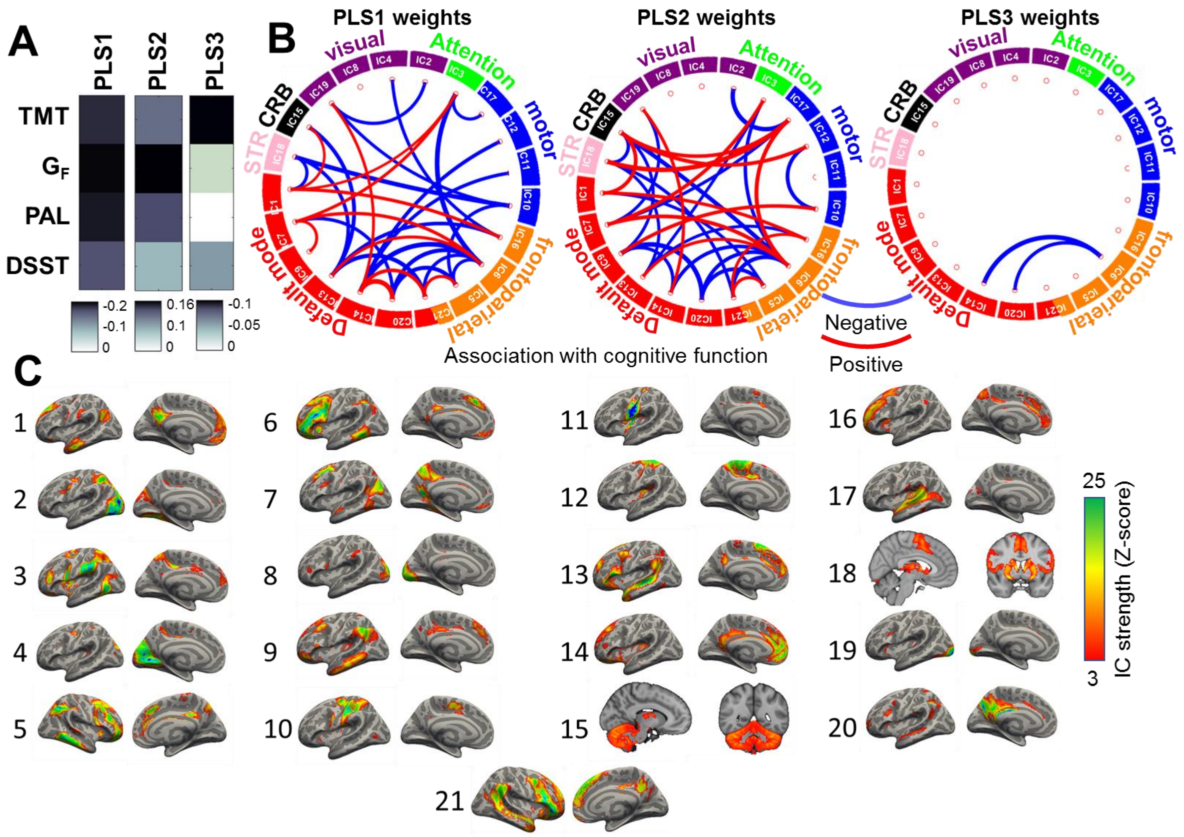

Supplementary Figure S11. Dimensional relationships between functional connectivity and executive function from a partial least squares (PLS) regression in healthy controls. Higher FC scores on PLS1 were associated with worse executive function, while higher FC scores on PLS2 were associated with better executive function (A). Thresholded PLS1 and PLS2 weights implicated pairwise connectivities between independent components corresponding to the default mode (DMN), frontoparietal (FPN) and dorsal/ventral attention (DA/VA) networks (B). Blue connections between network components suggest that higher connectivity of those components was associated with worse cognitive performance. Red connections between network components suggest that higher connectivity of those components predicted better cognitive performance. Anatomical locations of the independent components are shown in (C). Network labels include: MOT – motor; CRB – cerebellum; VIS – visual; STR – striatum. TMT – Trailmaking path; Gf – fluid intelligence test; PAL – paired associates learning; DSST – digit-symbol substitution test.

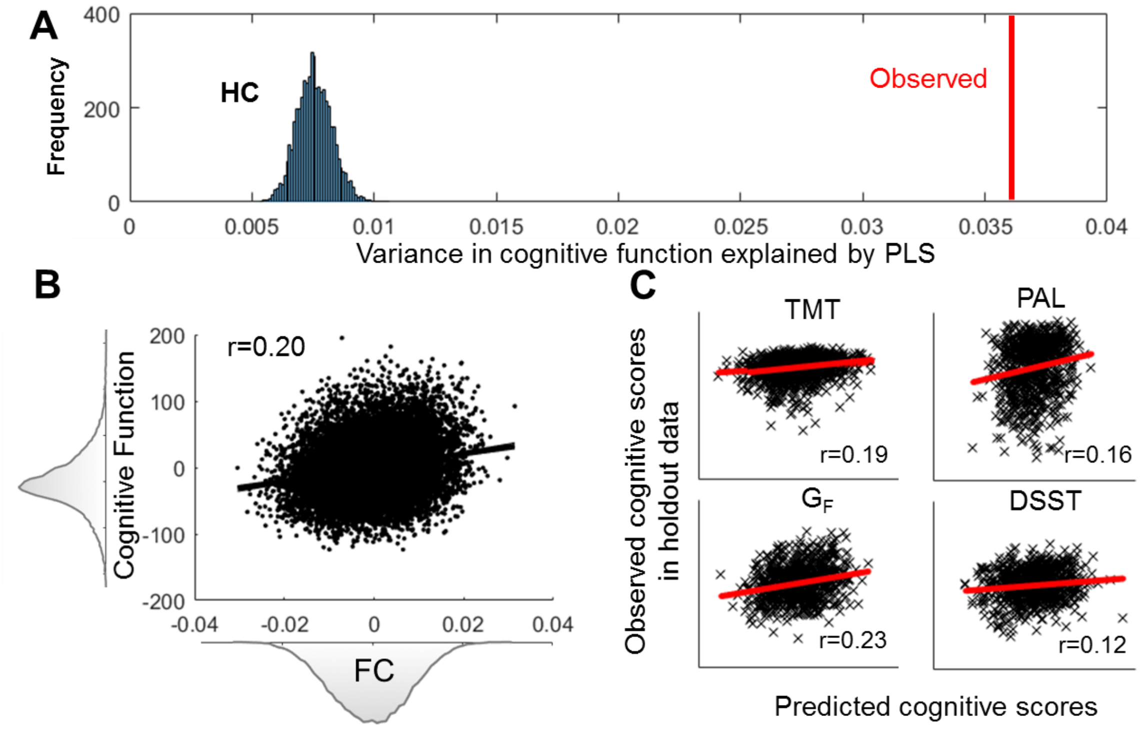

Supplementary Figure S12. Overview of the PLS findings in healthy controls. The PLS model explained a significantly greater amount of variance (3.6%) in cognitive function than expected by chance from a permutation distribution (A, shown in dark blue). The scores of the resulting latent variable PLS1, summarizing functional connectivity were predictive of PLS1 scores summarizing cognitive function in the full control sample (B). The PLS model achieved good performance in a hold-out analysis, with moderate correlations between predicted and observed cognitive scores in held-out data. (C).

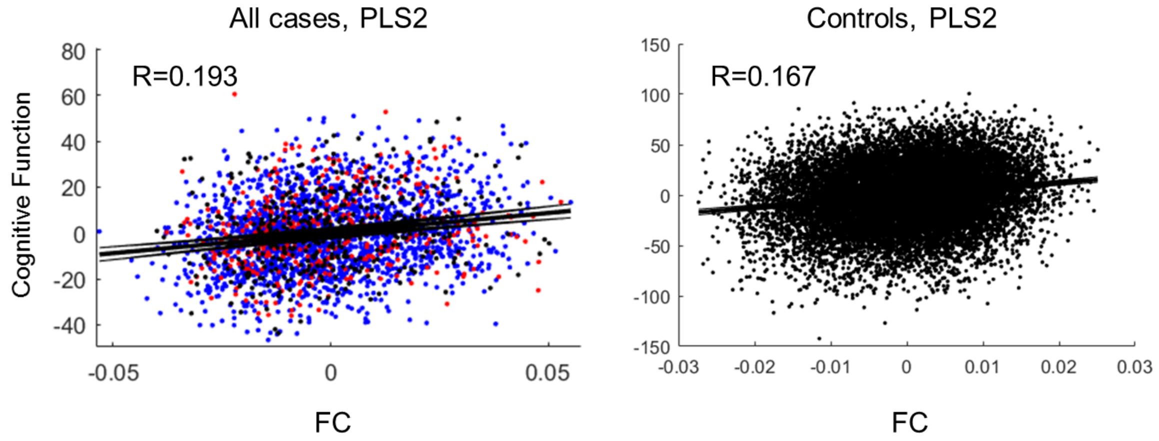

Figure S13. Correlation between PLS2 scores summarizing functional connectivity (FC) and PLS2 scores summarizing cognitive function in all cases (left) and in controls (right).

Supplementary Figure S14. Breakdown of sample sizes for each of the analyses. We pulled MRI data for 40,669 participants, of whom 31,291 participants were categorized as either HC, MDD- (Major depressive disorder), ANX- (non-phobic anxiety disorders), MDD+ANX, and STR- (stress-related disorders). Some of these participants were missing either cortical thickness, resting-state fMRI (due to quality issues) or cognitive data. Different patterns of missingness were found for cognitive tests. We also show the sample sizes for participants with both fMRI and cognitive data, which formed the basis of the brain-cognition analyses. TMT: Trailmaking; Gf: fluid intelligence; PAL: paired associate learning; DSST: digit-symbol substitution.

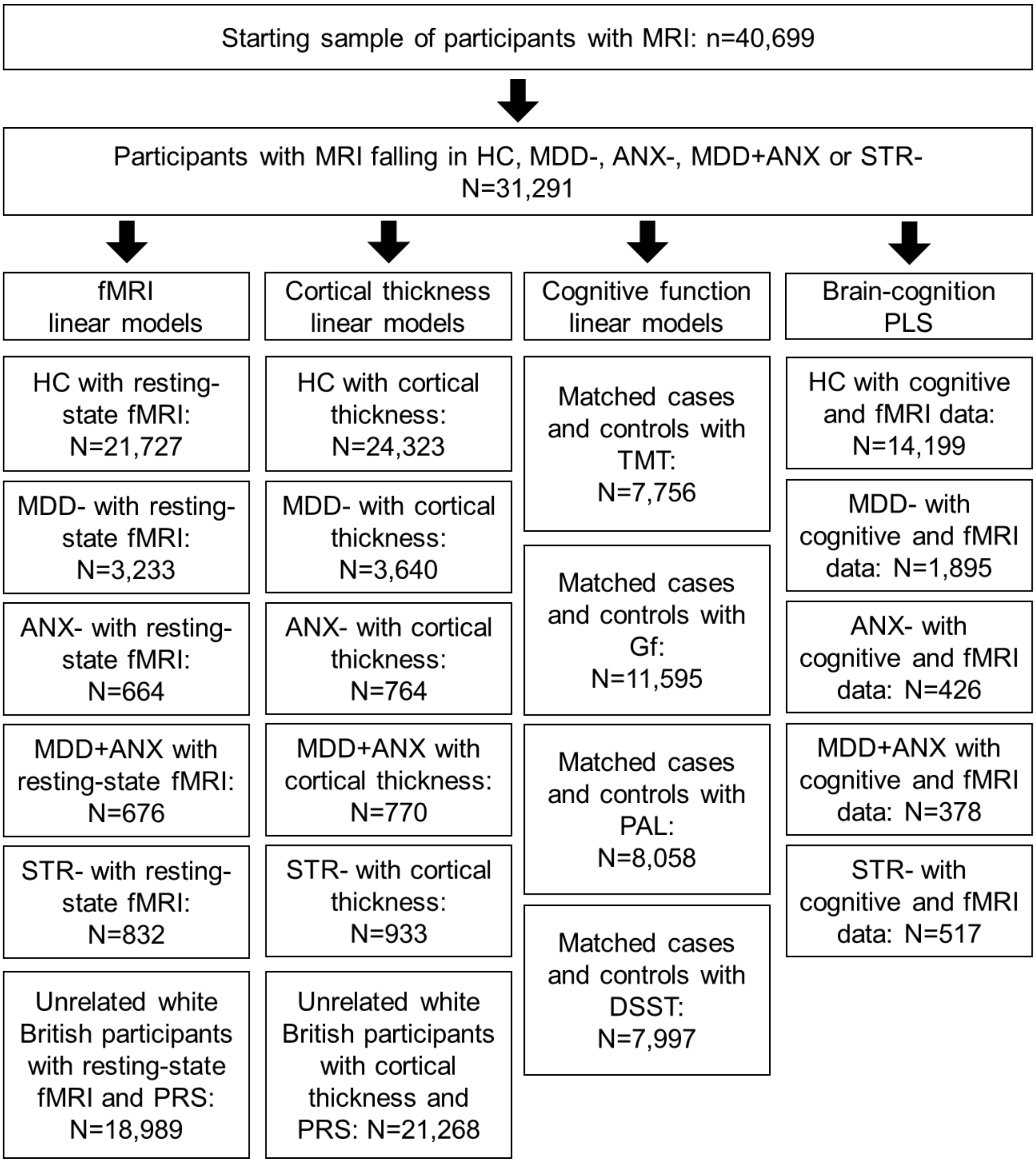

Medication Information

Supplementary Table 5. Medications included in each of the medication classes. SSRI, selective serotonin reuptake inhibitor; SARI: Serotonin antagonist and reuptake inhibitors; SNRI, selective noradrenaline (or adrenaline) reuptake inhibitor; NaSSA: Noradrenergic and specific serotonergic antidepressants; TCA: tricyclic antidepressants; MAO-I, monoamine oxidase inhibitors; Atypical – atypical antidepressants.

Medication	Class	Category
Citalopram	SSRI	1
Fluoxetine	SSRI	1
Sertraline	SSRI	1
Paroxetine	SSRI	1
Escitalopram	SSRI	1
Fluvoxamine	SSRI	1
Vortioxetine	SSRI	1
Trazodone	SARI	1
Nefazodone	SARI	1
Amitriptyline	TCA	2
Dosulepin	TCA	2
Lofepramine	TCA	2
Clomipramine	TCA	2
Nortriptyline	TCA	2
Imipramine	TCA	2
Trimipramine	TCA	2
Doxepin	TCA	2
Maprotiline	TCA	2
Protriptyline	TCA	2
Amoxapine	TCA	2
Phenelzine	MAOI	3
Moclobemide	MAOI	3
Tranylcypromine	MAOI	3
Isocarboxazid	MAOI	3
Venlafaxine	SNRI	4
Duloxetine	SNRI	4
Reboxetine	NRI	4
Mianserin	NaSSA	5
Mirtazapine	NaSSA	5

1. Cheng ST, Chan ACM, Yu ECS (2006) An exploratory study of the effect of mahjong on the cognitive functioning of persons with dementia. *Int J Geriatr Psychiatry* 21(7):611–617.

2. Maia PD, Kutz JN (2017) Reaction time impairments in decision-making networks as a diagnostic marker for traumatic brain injuries and neurological diseases. *J Comput Neurosci* 42(3):323–347.

3. Schwartz F, et al. (1989) Reaction time impairment in schizophrenia and affective illness: The role of attention. *Biol Psychiatry* 25(5):540–548.

4. Váša F, et al. (2018) Adolescent tuning of association cortex in human structural brain networks. *Cereb Cortex* 28(1):281–294.

5. Alexander-Bloch AF, et al. (2018) On testing for spatial correspondence between maps of human brain structure and function. *Neuroimage* 178:540–551.

6. Miller KL, et al. (2016) Multimodal population brain imaging in the UK Biobank prospective epidemiological study. *Nat Neurosci* 19(11):1523–1536.

7. Levis B, et al. (2020) Accuracy of the PHQ-2 Alone and in Combination with the PHQ-9 for Screening to Detect Major Depression: Systematic Review and Meta-analysis. *JAMA - J Am Med Assoc* 323(22):2290–2300.
